# Revision of the new world genus *Crassomicrodus* Ashmead (Hymenoptera, Braconidae, Agathidinae), with an identification key to species

**DOI:** 10.3897/zookeys.142.1709

**Published:** 2011-10-31

**Authors:** José Isaac Figueroa, Michael Joseph Sharkey, Jesus Romero Nápoles, José Antonio Sánchez García, Ana Mabel Martínez, Victor López-Martínez, Samuel Pineda

**Affiliations:** 1 Instituto de Investigaciones Agropecuarias y Forestales, Universidad Michoacana de San Nicolás de Hidalgo, Km. 9.5 carretera Morelia-Zinapecuaro, Tarímbaro, Michoacán, 58880, México; 2 Department of Entomology, University of Kentucky, S-225 Ag. Science Center North, Lexington, Kentucky 40546-0091, USA; 3 Instituto de Fitosanidad, Colegio de Postgraduados, Km. 36.5 Carretera México-Texcoco, Montecillo Edo. de México, 56230, México; 4 CIIDIR-IPN-Unidad Oaxaca, Area de Control biológico, Hornos #1003, Santa Cruz xoxocotlán, Oaxaca, C.P. 71230, México; 5 Facultad de Ciencias Agropecuarias, Universidad Autónoma del Estado de Morelos, Av. Universidad 1001, Col. Chamilpa, Cuernavaca, Morelos, C.P. 62210, México

**Keywords:** insecta, taxonomy, parasitoid wasps, new species, Ichneumonoidea

## Abstract

A key to species and descriptions are presented for 14 species of the New World genus *Crassomicrodus* Ashmead. Seven new species, *Crassomicrodus azteca*, *Crassomicrodus clypealis*, *Crassomicrodus costaricensis*, *Crassomicrodus jalisciensis*, *Crassomicrodus mariae*, *Crassomicrodus oaxaquensis*,and *Crassomicrodus olgae* are described. *Crassomicrodus fenestratus* (Viereck) is synonymized with *Crassomicrodus nigriceps* (Cresson). *Crassomicrodus melanopleurus* (Ashmead) is recognized as a valid species.

## Introduction

The agathidine wasp genus *Crassomicrodus* was erected by Ashmead (1900), with the type species *Microdus fulvescens* (Cresson, 1865). A few years later Bradley (1916) pointed out that *Microdus divisus* (Cresson, 1873), the type species of *Epimicrodus* (Ashmead 1900), shared many characters with the designated type of *Crassomicrodus*, and therefore synonymized *Epimicrodus* Ashmead under *Crassomicrodus*. Members of *Crassomicrodus* are characterized by a short ovipositor, tarsal claws without basal lobes, and the lack of pegs near the apex of the lateral surface of the hind tibia. *Crassomicrodus* is closely related with the genus *Agathirsia* Westwood (Pucci and Sharkey 2004). The original placement of *Crassomicrodus* and *Agathirsia* in the tribe Agathidini was proposed by Sharkey (1992) but recently Sharkey et al. (2006), using both molecular and morphological data sets, proposed the tentative inclusion of these genera in the tribe Earinini.

Prior to this publication *Crassomicrodus* contained eight species recognized (Muesebeck et al. 1951, Shenefelt 1970, Marsh and Carlson 1979, Figueroa et al. 2008). Previous to 2008 there were nine nominal species but Figueroa et al. (2008) synonymized *Crassomicrodus medius* Cresson under *Crassomicrodus fulvescens*. Six species were included in the Hymenoptera of America North of Mexico (Muesebeck 1927). One species was described from Puerto Rico (Viereck 1913), and Marsh (1960) added one species collected in the U.S.A. Other nominal species, such as *Crassomicrodus nigricaudos* (Viereck 1905) and *Crassomicrodus pumilus* = *Epimicrodus pumilus* (Szépligeti 1913, Brues 1926) were included in the genus, but these were misidentified. *Crassomicrodus nigricaudos* was transfered to *Agathirsia* by Muesebeck (1927), and *Epimicrodus pumilus* was transferred to the genus *Bassus* by Pucci and Sharkey (2004). According to Sharkey (1997) species of *Crassomicrodus* are restricted to the New World, and can be found from southern of Canada to Costa Rica, although in this study we found one species from Colombia. *Autographa californica* (Speyer), a lepidopteran, is the unique host record for *Crassomicrodus*, i.e., *Crassomicrodus fulvescens* (Cresson) (Sharkey 1997, Figueroa et al. 2008).

In this revision, we redescribe seven species, describe seven new species of *Crassomicrodus*, and synonymize *Crassomicrodus nigriceps* under *Crassomicrodus fenestratus*. We also recognize *Crassomicrodus melanopleurus* as a valid species and provide an identification key for all species of *Crassomicrodus*.

## Materials and methods

**Species treatments.** Descriptions of all included species are based on all material examined. All measurements were performed using a micrometer adapted to an Iroscope microscope and are given in millimeters. Terminology used for the species descriptions follows Sharkey and Wharton (1997) and Sharkey (2006) and for microsculpture of surface we follow Eady (1968) and Sharkey and Wharton (1997). Data labels were transcribed to a database in the Program Paradox Version 4.5 and the information is presented in a standardized format organized by country and state or province. All photographs were taken using a Leica MZ 16 stereoscope equipped with JVC KY-F75 3CCD digital camera and were prepared using an Auto-Montage imaging system.

**Specimens sources.** For this revisión were borrowed the types of *Crassomicrodus apicipennis* Muesebeck, *Crassomicrodus divisus* (Cresson), *Crassomicrodus fenestratus* Viereck, *Crassomicrodus fulvescens* (Cresson), *Crassomicrodus medius* (Cresson), *Crassomicrodus muesebecki* Marsh, *Crassomicrodus nigriceps* (Cresson), *Crassomicrodus nigrithorax* Muesebeck, *Crassomicrodus pallens* (Cresson), *Microdus melanopleurus* Ashmead and *Orgilus rileyi* Ashmead. Paratypes, homotypes and additional specimens were provided from the following institutions: American Entomological Institute Collection, Florida (AEIC); American Museum of Natural History, New York (AMNH); Academy of Natural Sciences, Philadelphia, Pennsylvania (ANSP); California Academy of Sciences, San Francisco (CAS); Universidad Autónoma de Nuevo León, Nuevo León (CIBE-UANL); Canadian National Collection, Ottawa (CNC); Cornell University Insect Collections, New York (CUIC); Essig Museum of Entomology, University of California, California (EMEC); University of Wyoming, Wyoming (ESUW); Florida State Collection of Arthropods, Florida (FSCA); Hymenoptera Institute Collection, University of Kentucky, Kentucky (HIC); Instituto de Biología, Universidad Autónoma de México (IBUNAM); Fundación e Instituto Miguel Lillo, Universidad Nacional de Tucumán, Argentina (IMLA); Illinois Natural History Survey, Illinois (INHS); Instituto Nacional de Investigaciones Forestales Agrícolas y Pecuarias, Guanajuato (INIFAP); University of Wisconsin, Wisconsin (IRCW); Kansas State University Collection, Kansas (KSUC); Museum of Comparative Zoology, Harvard University (MCZ); Michigan State University Collection, Michigan (MSUC); Museo de la Universidad de Costa Rica (MUCR); Ohio State University, Ohio (OSU); Texas A & M University, Texas (TAMU); Universidad Autónoma de Yucatán (UADY); The Bohart Museum of Entomology, University of California-Davis, California (UCDC); University of Colorado Museum, Colorado (UCMC); University of California-Riverside, California (UCR); Enns Entomology Museum, University of Missouri-Columbia, Missouri (UMRM); University of Minnesota-St. Paul, Minnesota (UMSP); Smithsonian National Museum of Natural History, Washington (USNM).

## Descriptions and keys

### Genus Crassomicrodus

Ashmead, 1900

http://species-id.net/wiki/Crassomicrodus

Crassomicrodus Ashmead 1900. Type species *Microdus fulvescens*Cresson 1865, designated by Ashmead 1900 [Examined].
Epimicrodus Ashmead 1900. Type species *Microdus divisus*Cresson 1873, designated by Ashmead 1900 [Examined].
Crassomicrodus Ashmead= (*Epimicrodus* Ashmead) synonymized by Bradley 1916.

#### Diagnosis.

*Crassomicrodus* species can be distinguished from other agathidines with the following combination of characters: simple tarsal claws, without basal lobes, apicolateral pegs of the hind tibia are hair-like, labio-maxillary complex not elongate; mandible with two teeth; and metasomal tergum 1 smooth.

#### Description.

Head. Transverse or triangular; area between antennal sockets with a median pyramidal-shaped elevation or transverse; gena not bulging to distinctly bulging; labio-maxillary complex not elongate, mandible with two teeth, antenna with 25 to 43 flagellomeres.

#### Mesosoma.

 Pronotum surface smooth or punctuate; notauli from lacking to impressed; anterolateral edges of scutellum with or without a small acute projection; lateral scutellar depression from smooth to crenulate; dorsal surface of propodeum from rugulose to reticulate rugose; subalar lobe separated from mesopleuron by a wide or narrow groove; metapleuron from smooth to reticulate rugose; inner spur of hind tibia from 0.47 to 0.78 times longer than basitarsus; tarsal claw without basal lobe; outer apex of the hind tibia without flattened pegs; forewing vein R1 0.47–0.70 times longer than RS; crossvein *r* arising before or beyond middle of stigma. Metasoma. Metasomal median tergite 1 smooth; apical width 1.78–3.92 times longer than basal width; ovipositor sheaths and ovipositor variable in length; metasoma 1.00–1.42 times longer than mesosoma.

##### Key to the New World species of Crassomicrodus Ashmead

**Table:** 

1	Head triangular in frontal view (Figs 2a, 4a, 5a, 6a, 10a, 12a, 14a); gena not bulging; area between antennal sockets with a median pyramidal-shaped elevation (Figs 2a, 4a, 5a, 6a, 10a, 12a, 14a); length of ventrolateral margin of clypeus similar to diameter of tentorial pit (Fig. 1a, 8a)	2
–	Head transverse in frontal view (Figs 1a, 3a, 7a, 8a, 9a, 11a, 13a), if somewhat triangular then length of ventrolateral margin of clypeus longer than diameter of tentorial pit (Fig. 3a); gena bulging or at least slightly bulging (Figs 1a, 3a, 7a, 8a, 9a, 11a, 13a); area between antennal sockets variable in shape	10
2(1)	Malar space at least 0.8 times as long as eye height (Figs 5a, 14a)	3
–	Malar space at most 0.6 times as long as eye height (Figs 2a, 4a, 6a, 10a, 12a)	4
3(2)	Scutellar sulcus with 3 or 4 carinae; second submarginal cell quadrangular; fore and middle legs black; body length 6.95–8.60 mm	*Crassomicrodus divisus* (Cresson)
–	Scutellar sulcus with 1 carina; second submarginal cell triangular; fore and middle legs yellowish-orange; body length 4.20–6.48 mm	*Crassomicrodus pallens* (Cresson)
4(2)	Forewing vein R1 at most half the length of RS; head yellowish-orange (Fig. 14a)	*Crassomicrodus pallens* (Cresson)
–	Forewing vein R1 at least 0.6 times longer than RS; head black (Fig. 4a)	5
5(4)	Hind wing vein 1M less 1.0 times as long as 1r-m; body black; wings strongly infumate (Fig. 4e); body length at least 8.1 mm	*Crassomicrodus costaricensis* sp. n.
–	Hind wing vein 1M at least 1.4 times as long as 1r-m; body at least with some areas yellowish-orange or yellowish-red (Figs 2d, 6e, 12e), if body dark then body length less than 7.9 mm; wings at most slightly infumate (Fig. 10e)	6
6(5)	Head and mesosoma black (Figs 2d, 12e); posterior surface of antennal sockets at least slightly rugulose	7
–	Head and mesosoma usually with some areas yellowish-orange or yellowish-red (Figs 6cde, 10cde), if entirely black then the posterior surface of antennal sockets smooth (Fig. 10b)	9
7(6)	Subalar lobe separated from mesopleuron by a wide groove (Fig. 6c); body length 7.10–7.65 mm; 38–40 flagellomeres	8
–	Subalar lobe separated from mesopleuron by a narrow groove (Fig. 2c); body length 5.20–5.50 mm; 31–33 flagellomeres	*Crassomicrodus azteca* sp. n.
8(7)	Hind legs black (Fig. 12e); wings hyaline; posterior area of antennal sockets rugulose (Fig. 12b)	*Crassomicrodus oaxaquensis* sp. n.
–	Hind femora yellowish-orange (Fig. 6e); wings slightly infumate; posterior area of antennal sockets rugose (Fig. 6b)	*Crassomicrodus jalisciensis* sp. n.
9(6)	Setae length on body surface slightly similar to setae length at base of mandible (6ac); posterior surface of antennal sockets rugose (Fig. 6b); frons deeply excavated; pronotum punctulate (Fig. 6c); propodeum reticulate rugulose	*Crassomicrodus jalisciensis* sp. n.
–	Setae length on body surface distinctly smaller than setae length at base of mandible (Figs 10ac); posterior surface of antennal sockets smooth (Fig. 10b); frons excavated; pronotum smooth (Fig. 10c); propodeum rugose	*Crassomicrodus nigriceps* (Cresson)
10(1)	Area between antennal sockets with a median pyramidal-shaped elevation (Figs 3a, 7a)	11
–	Area between antennal sockets at least weakly transverse or with a median elevation in trapezoidal shape (Figs 1a, 8a, 9a, 11a, 13a)	14
11(10)	Frons at least slightly excavated (Figs 3ab, 8ab); notauli impressed (Figs 3d, 8d); forewing M+CU pigmented over most its length; 34–41 flagellomeres	12
–	Frons not excavated (Figs 7ab); notauli not impressed (Fig. 7d); forewing M+CU unpigmented over most its length; 25–28 flagellomeres* C. mariae* sp. n.
12(11)	Length of ventrolateral margin of clypeus similar to diameter of tentorial pit (Fig. 8a)	13
–	Length of ventrolateral margin of clypeus distinctly longer than diameter of tentorial pit (Fig. 3a)	*Crassomicrodus clypealis* sp. n.
13(12)	Inner spur of hind tibia distinctly longer than half of basitarsus length (0.60–0.72); gena bulging (Fig. 8a); lateral depression of scutellum smooth, only its ventral edge with small punctures	*Crassomicrodus melanopleurus* Ashmead
–	Inner spur of hind tibia less than half length of basitarsus (a few specimens with the spur at most reaching 0.54 times); gena distinctly bulging; lateral depression of scutellum rugose and fovelate	*Crassomicrodus fulvescens* (Cresson)
14(10)	Head and mesosoma black; anterolateral edges of scutellum lacking small acute projection	15
–	Head and mesosoma at least with some areas yellowish-orange or yellowish-red (Figs 1acde); anterolateral edges of scutellum with small acute projection (Fig. 1df)	*Crassomicrodus apicipennis* Muesebeck
15(14)	Notauli not impressed over most of mesoscutum (Fig. 9c); metasoma black; setae at base of mandible similar in size to setae on rest of body surface (9ad); length of setae in scutellar disk 0.18 to 0.20 mm; ovipositor sheaths of female at least 1.83 mm in length	*Crassomicrodus muesebecki* Marsh
–	Notauli impressed over most of mesoscutum (Fig. 11c, 13d); metasoma yellowish-orange (Figs 11e, 13e); setae at base of mandible distinctly longer than setae on rest of body surface (11ad, 13ac); length of setae in scutellar disk less than 0.17 mm; ovipositor sheaths of female at most 0.22 mm in length	16
16(15)	Area between antennal sockets with a median elevation in trapezoidal shape (Fig. 13a); gena distinctly bulging (Fig. 13a); malar space 0.54–0.59 times longer than eye height; 31–34 flagellomeres; body length 6.70–7.08 mm	*Crassomicrodus olgae* sp. n.
–	Area between antennal sockets with a median transverse elevation (Fig. 11a); gena bulging (Fig. 11a); malar space 0.38–0.47 times longer than eye height; 28–31 flagellomeres; body length 3.95–5.35 mm	*Crassomicrodus nigrithorax* Muesebeck

##### Species descriptions of Crassomicrodus

### Crassomicrodus apicipennis

Muesebeck, 1927

http://species-id.net/wiki/Crassomicrodus_apicipennis

Fig. 1a–f

Crassomicrodus apicipennis Muesebeck 1927: 18–19

#### Holotype female.

 Mount Hood, Oregon [USA]. Cat. No. 28695 (USNM).

#### Description female.

**Figure 1.**
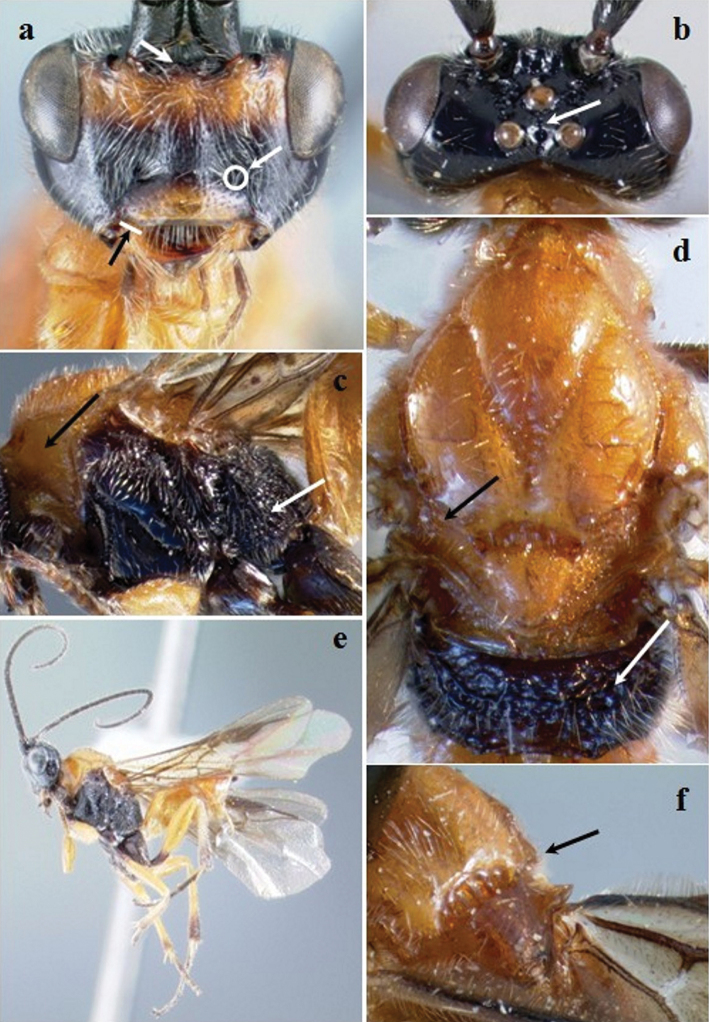
*Crassomicrodus apicipennis*. Female **a** anterior view of head, arrow indicates a median transverse elevation with two weakly defined lateral tubercles, ventrolateral margin of clypeus, and tentorial pit **b** dorsal view of head, arrow indicates groove between lateral ocelli with small foveolae **c** lateral view of mesosoma, arrows indicate pronotum and metapleuron **d** dorsal view of mesosoma, arrows indicate anterolateral edge of scutellum and propodeum **e** female habitus **f** dorsal view of scutellum, arrow indicates anterolateral edge of scutellum with a small acute projection.

Body. Length. 4.90–5.97 mm. Color (Fig. 1e). Integument black except yellowish-orange as follows, basal area of mandible, pronotum, mesonotum, subalar lobe, tegula, metasoma, femora, basal area of hind tibia, anterior and middle tibia and tarsomeres; mandible apex, basal area of hind tibia; tarsomeres blackish; wing veins dark brown; forewing infumate with a hyaline spot on the first submarginal cell that is similar in size to the parastigma. Specimens range from black to yellowish-orange on the head, propleuron, metanotum, propodeum, mesopleuron, metapleuron and hind coxa. Head (Fig. 1ab). Transverse in frontal view; face dorsomedially with weak longitudinal ridge in most specimens; eye height/width = 1.31–1.35; eye height 0.65–0.68× inter-ocular distance; area between antennal sockets with a median transverse elevation and two weakly defined tubercles; frons deeply excavated and rugulose with small foveolae; posterior surface of antennal sockets smooth; groove between lateral ocelli with small foveolae; median ocellus separated from lateral ocellus by groove with small foveolae; gena bulging; malar space 0.48–0.55× as long as eye height; clypeus (anterior view) 2.29–2.33× wider than high; length of ventrolateral margin of clypeus similar to diameter of tentorial pit; antenna with 29–32 flagellomeres; setae at base of mandible distinctly longer than setae on rest of body surface. Mesosoma (Fig. 1c–f). Pronotum smooth; lateral pronotal margins with weakly crenulate groove; notauli impressed; anterolateral edges of scutellum with a small acute projection; scutellar disc convex with sparse setae from 0.16 to 0.17 mm in length; scutellar disc sloped posteriorly and rounded; lateral scutellar depression rugose and foveolate; carinae of central metanotal area forming a triangular cell; propodeum reticulate rugose, more pronounced on lateral margins; subalar lobe separated from mesopleuron by narrow rugulose groove, width distinctly of shorter than subalar lobe; metapleuron rugose with reticulate-foveolae. Legs. Inner spur of middle tibia 0.76–0.83× length of basitarsus; inner spur of hind tibia 0.59–0.69× length of basitarsus; metabasitarsus 1.18–1.25× length of tarsomeres III, IV, and V combined; hind tibia 2.00–2.21× longer than basitarsus; hind femur length 3.38–4.12× its maximum width. Wings. Forewing length/width = 2.46–2.51; stigma 2.69–3.50× longer than maximum width; forewing vein R1 0.60–0.66× as long as vein RS; vein RS sinuate; vein r arising slightly before middle of stigma; second submarginal cell triangular, with petiole 0.07–0.13 mm long; vein M+CU distinctly pigmented throughout; hind wing length/width = 3.37–3.52; hind wing vein 1M 1.65–1.80× longer than 1r-m; hind wing with 4–5 hamuli. Metasoma. Apical width of petiole (tergum 1) 2.90–3.21× wider than basal width; minimum width of petiole 0.58–0.60× apical width; length of ovipositor sheath 0.17–0.30 mm.

#### Male.

Similar to female.

#### Host.

Unknown.

#### Distribution.

Canada, Mexico, and USA.

#### Diagnosis.

 Distinguished from other *Crassomicrodus* species by the following combination of characters: area between antennal sockets with a median transverse elevation, anterolateral edges of scutellum with a small acute projection, groove between lateral ocelli with small foveolae, and head and mesosoma black with some areas yellowish-orange.

#### Remark.

 This species is near to *Crassomicrodus nigrithorax*, but differs in that *Crassomicrodus nigrithorax* has the mesosoma black; anterolateral edges of scutellum lacking small acute projection; eye height 0.69–0.70× inter-ocular distance; malar space 0.38–0.47× longer than eye height; inner spur of middle tibia 0.89–0.95× length of basitarsus; and scutellar disc convex with sparse setae from 0.14 to 0.15 mm in length.

#### Material examined.

 Holotype ♀: USA, Oregon: Mt. Hood. Collection Ashmead (USNM). Allotype ♂: same data as type (USNM). Homotype ♀: USA, Iowa: Iowa Co., 10/VIII/1934, Jaques H.E. (CNC). *Other specimens examined*.- **CANADA, Alberta:** Alta. 1 ♀; Scandia Alta., 1 ♀ 26/VII/1956, Peck O. (CNC). **British Columbia:** Hedley, Nickel Plate City, 1♀ 17/VIII/1953, 1524 m, Martin J.E.H.; Kamloops 1 ♂ 9/VII/1939, Spencer G.J.; Richter Pass, Osoyoos 1 ♀ 28/VI/1959, Kelton L.A. (CNC). **Manitoba:** Aweme, N Criddle, 1 ♀ 9/VII/1924 (CNC). **MEXICO, Durango:** 5 mi. W Durango 1 ♂ 24/VI/1964, Howden H.F. (HIC). **Sonora:** Nogales 1 ♂ 4/IX/1965, Michelbacher A.E. (EMEC); **Tamaulipas:** Santa Teresa 1 ♂ 15/V/1952, Cazier M., W. Gertsch & R. Schrammel (AMNH). **USA:** Iowa Co., 1 ♀ 19/VI/1935, Huizinga H. (USNM). **Arizona:** Springerville, 1 ♂ 26/VII/1956, Butler-Gerhardt (USNM); 9 km SE Camp Verde, Clear Creek, Yavapai Co., 1 ♂ 11/IX/1986, Parker F. & T. Griswold (CNC). **Colorado:** 1 ♂; Boulder Canyon, 1 ♀ 8/VIII/1960, 2377 m, Dreisbach R.K. (USNM); 3 mi. NW Wiggins, Morgan Co., 1 ♂ 8/VIII/1974, Favreau M. & T.M.; Pagosa Springs, 1 ♀, 1 ♂ 22–24/VI/1919, 2195 m, 37.16 N 107.0 W (AMNH); Craig, 1 ♂ 25/VI/1949, Bryant L. (CAS); Fort Morgan, 1 ♂ 5/VIII/1960, Dreisbach R.K. ; Limon, 1 ♂ 16/VIII/1949, Dreisbach R.R. & R.K. Shwab (MSUC); Limon, 1 ♂ 16/VIII/1949, Dreisbach R.R. & R.K. Shwab (AEIC); Nunn, 1 ♀ 7/VI/1976, Lavigne R. (ESUW); Prowers Co., 1 ♀ 5/VI/1962, Marston N. (KSUC); Running Creek Field Sta. Elbert Co. T9S, R65W, Sec 35 NE nativo, 2 ♀ 2118 m, Brown F.M. (UCMC). **Kansas:** Manhattan, 1 ♀, 1 ♂ 16/VII/1950, Evans H.E.; Manhattan, 1 ♂ 24/V/1935, Wilbur D.A. (KSUC); Sitka, Clark Co., 1 ♀ 12/VI/1960, Eickwort G.C. (MSUC). **Minnesota:** Albert Lea, Freeborn Co., 1 ♀, 1 ♂ 11/VI/1961, Levi H. (MCZ); Browns Valley, 1 ♂ 4/VIII/1935, Denning D.G.; Lincoln Co., 1 ♂ 4/VI/1938, Mickel C.E.; Ortonville: 1 ♂ 5/VIII/1935, Deggy R.H.; 6 ♂ 5/VIII/1935, Denning D.G.; Polk Co., 1 ♀ 16/VIII/1936, Daggy R.H.; Yellow, Medicine Co., 1 ♂ 13/VIII/1936, Mickel C.E. (UMSP). **Missouri:** Diehlstadt, 1 ♂ 5/VIII/1938, Wingo C. (UMRM); Columbia, 1 ♂ 7/VIII/1958, Blickenstaff C.C. (USNM). **Nebraska:** Valentine Refuge, 1 ♂ 7/VI/1972, Townes H. & M. Townes (AEIC); 1.5 mi. N Mullen (Middle Loup River), Hooker Co., 1 ♀ 2–4/VII/1983, Grissell & Menke (HIC); Thomas Co., 1 ♂ 21/VIII/1951, Dreisbach R.R. (MSUC); Halsey: 1 ♂ 12/VIII/1925, Dawson R.W.; 1 ♂ 15/VIII/1925, Dawson R.W.; McCool, 1 ♀ 22/VIII/1940, Milliron H.E. (UMSP); Morril Co., 1 ♂ 22/VIII/1951, Dreisbach R.R. (USNM). **Oklahoma:** Lake Carl Etling, Black Mesa State Park, 25 mi. by road NW Boise City, Cimarron Co., 1 ♂ 14/VIII/1967, Leech H. (CAS); Pond Creek, Grant Co., 2 ♀ 11/VI/1960, Eickwort G.C. (MSUC). **Texas:** Spring, 1 ♂ 21/VI/1947, Rockefeller D. (AMNH); 30 mi. N Uvalde, Uvalde Co., 1 ♀ 21/VI/1983, Pulawski W.J. (CAS); East Hwy. side of Road way park, 7 mi. S Beeville, 1 ♀ 2/VI/1964, Hull F.M. & M.C. (CNC); Sam Houston, Bexar Co., 1 ♀ 31/III/1953, Adelson B.J. (EMEC); 3 mi. W Estelline, Hall Co., 1 ♀ 3/VI/1979, Michener C.D. (HIC);Alice: 1 ♂ 17/VII/1954, Dreisbach R.R. (MSUC); 1 ♂ 17/VII/1954, Dreisbach R.R. (USNM); Bangs, 1 ♀ 6/VIII/1938 (USNM); Bentsen Rio Grande Valley State Park. Hidalgo Co.: 1 ♀ 11/VIII/1983, Bars M. Kaul (CNC); 1 ♂ 10/VI/1982, 1 ♂ 10/VIII/1983, 1 ♂ 11/VI/1982, 1 ♂ 15/VIII/1983, 1 ♂ 16/VIII/1983, 1 ♂ 25/V/1977, 1 ♂; 27/VI/1983, 1 ♂ 28/VI/1983, 3 ♀ 4/VI/1982, 1 ♂ 9/VI/1982, Porter C. (FSCA); Bentsen Rio Grande Valley State Park. Nr. Mission, Hidalgo Co.: 1 ♀, 1 ♂ 13/VI/1983, 1 ♀, 1 ♂ 20/VI/1983, 1 ♂ 22/III/1984, 1 ♂ 26/VII/1984, 1 ♀ 30/VIII/1983, 4 ♀, 2 ♂; 4/VIII/1983, Porter C. (FSCA); Dumas, Moore Co., 3 ♂ y 1 ♂ 14/VI/1960, Fischer R.L. (MSUC); Brownsville, 1 ♂ 6/VII/1895; Kerrville, 1 ♀ 12/IV/1907, Pratt F.C.; College State, 2 ♂ 29/III/1954, Lewis W.J. (USNM). **Utah:** Goblin Vly, sand dunes, Emery Co., 1 ♀ 20/VI/1980, Parker F.D.; Wild Horse cr. W Goblin Vly., Emery Co., 1 ♀ 26/VII/1983, 1463 m, Parker F. & T. Griswold (CNC). Bonanza, Uintah Co., 1 ♀ 27/VI/1978, Bohart G. (UCMC). **Wyoming:** Glendo, 1 ♂ 24/VI/1960, Lavigne R.J. (USNM).

### Crassomicrodus azteca

Figueroa, Romero & Sharkey sp. n.

urn:lsid:zoobank.org:act:D1F7CD97-570A-42DB-B164-312E6869C770

http://species-id.net/wiki/Crassomicrodus_azteca

Fig. 2a–d

#### Description female.

Body. Length. 5.20–5.50 mm. Color (Fig. 2d). Integument black except yellowish-orange as follows, medial area of mandible, tegula, femora, anterior and middle tibiae and tarsomeres, and metasoma; eyes silver; ocelli translucent honey yellow; wing veins dark brown; forewing slightly infumate with a hyaline spot on the first submarginal cell that is similar in size to the parastigma. In some specimens the first metasomal tergite blackish. Head (Fig. 2ab). Triangular in frontal view; face with weak longitudinal ridge dorsomedially; eye height/width = 1.43–1.45; eye height 0.59–0.63× inter-ocular distance; area between antennal sockets with a median pyramidal-shaped elevation; frons excavated with a pair of microfoveolate grooves that diverge towards the ocellar area; posterior surface of antennal sockets slightly rugulose; groove between lateral ocelli smooth; median ocellus separated from lateral ocellus by smooth groove; gena not bulging; malar space 0.60–0.63× as long as eye height; clypeus 2.27–2.35× wider than high; length of ventrolateral margin of clypeus similar to diameter of tentorial pit; antenna with 31–33 flagellomeres; setae at base of mandible slightly longer than setae on rest of body surface. Mesosoma (Fig. 2bcd). Pronotum punctulate with setae; lateral pronotal margins with a shallow, crenulate groove; notauli impressed; anterolateral edges of scutellum lacking small acute projection; scutellar disc convex with sparse setae from 0.13 to 0.14 mm in length; scutellar disc sloped posteriorly and rounded; lateral scutellar depression smooth, sometimes with microfoveolate grooves in its ventral and dorsal margins; carinae of central metanotal area almost circular shaped, sometimes triangular; propodeum reticulate rugose with abundant setae in its lateral areas; subalar lobe separated from mesopleuron by narrow rugulose groove, width distinctly of smaller size than the subalar lobe; metapleuron reticulate rugulose in its ventral half and smooth in its dorsal half. Legs. Inner spur of middle tibia 0.65–0.70× length of basitarsus; inner spur of hind tibia 0.58–0.64× length of basitarsus; metabasitarsus 1.05–1.16× length of tarsomeres III, IV, and V combined; hind tibia 2.41–2.67× longer than basitarsus; hind femur length 3.50–4.00× its maximum width. Wings. Forewing length/width = 2.50–2.55; stigma 3.00–3.23× longer than maximum width; forewing vein R1 0.61–0.68× as long as vein RS; vein RS slightly straight; vein r arising before middle of stigma; second submarginal cell triangular, with petiole 0.07–0.12 mm long; vein M+CU weakly pigmented in 0.75 of its basal length; hind wing length/width = 3.37–3.38; hind wing vein 1M 1.44–1.70× longer than 1r-m; hind wing with 4–5 hamuli. Metasoma. Apical width of petiole (tergum 1) 3.46–3.71× wider than basal width; minimum width of petiole 0.53–0.59× apical width; length of ovipositor sheath 0.13–0.28 mm.

#### Male.

Similar to female, but the posterior surface of antennal sockets smooth.

#### Host.

*Copitarsia* sp. (Lepidoptera: Noctuidae) in *Brassica oleraceae* L. (cauliflower).

**Figure:**
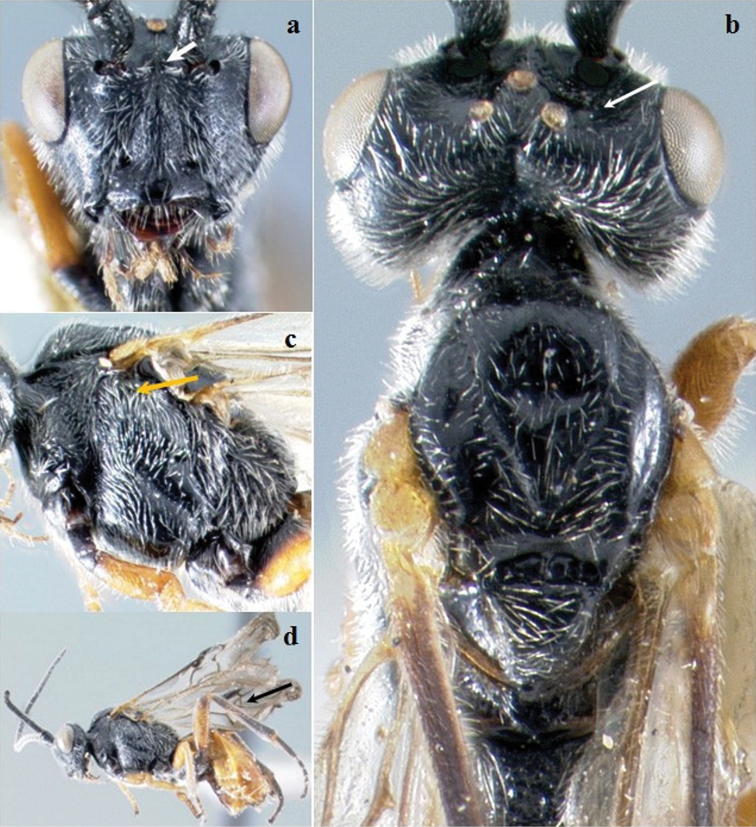
**Figure 2.** *Crassomicrodus azteca* sp. n.Female **a** anterior view of head, arrow indicates a median pyramidal-shaped elevation **b** dorsal view of head and mesosoma, arrow indicates posterior surface of antennal sockets slightly rugulose **c** lateral view of mesosoma, arrow indicates subalar lobe separated from mesopleuron by narrow groove **d** female habitus, arrow indicates triangular-shaped second submarginal cell.

#### Distribution.

Mexico.

#### Diagnosis.

 Distinguished from other *Crassomicrodus* species by the following combination of characters: area between antennal sockets with a median pyramidal-shaped elevation, malar space 0.60–0.63× as long as eye height, setae at base of mandible slightly longer than setae on rest of body surface, body length 5.20–5.50 mm, head and mesosoma black, and forewing slightly infumate.

#### Remarks.

This species is similar to *Crassomicrodus nigrithorax*, but differs in that *Crassomicrodus nigrithorax* has gena bulging; area between antennal sockets with a median transverse elevation; groove between lateral ocelli with small foveolae; and malar space 0.38–0.47× longer than eye height.

#### Etymology.

The specific epithet is a noun in apposition to *Crassomicrodus*, which is reference to some of the indigenous people of Mexico.

#### Material examined.

 Holotype ♀: MEXICO, México: Chapingo, 31/VII/1967, J.L. Carrillo Sánchez. *Copitarsia* sp. Allotype ♂: same data as holotype. Holotype and Allotype deposited in Instituto de Investigaciones Agropecuarias y Forestales, Universidad Michoacana de San Nicolás de Hidalgo, Tarímbaro, Michoacán, México (IIAF-UMSNH). Paratypes: **MEXICO, Guanajuato:** 10 mi. NW Leon, 1 ♂ 19/VIII/1954, 2042 m, Chillcott J.G. (CNC); Guanajuato, 1 ♀ 15/VIII/1953, Vaurie C. & P. Vaurie (AMNH). **Hidalgo:** Pachuca, Junction Rt. 85, 2 ♀ 24/IV/1965, 2438–2591 m, Weems H.V. Jr. (FSCA). **Jalisco:** Lagos de Moreno, 1 ♀ 12/VIII/1954, 1920 m, Dreisbach R.R. (USNM); Rancho La Quinta Teocaltiche, 1 ♂ 25/VIII/1979, 1707 m, Villegas B. (UCDC). **México state:** Chapingo: 1 ♂ 1950, F. Pacheco M. (USNM); 2 ♀ 1/VIII/1967, 7/VIII/1967, J.L. Carrillo Sánchez. *Copitarsia* sp., (IIAF); Chimalhuacán, 1 ♂ 28/VIII/1967, J.L. Carrillo Sánchez, *Copitarsia* sp. (IIAF); Pirámides de Teotihuacan, 3 ♀ 7/VII/1951, Hurd P.D. (USNM); Texcoco, 1 ♂ 12/VIII/1954, 2134 m, Chillcott J.G. (CNC). **Nuevo Leon:** 32 km W Linares, San Pedro Iturbide, 1 ♂, 2 ♀ 5/X/1962, Townes H. & M. Townes (AEIC). **Puebla:** 14 km NE Cañada Morelos, 1 ♂ 10/VII/1974, Chemsak J., E.G. Linsley & J. Linsley (EMEC). **Zacatecas:** 15 km E Sombrerete, 1 ♂ 30/VII/1951, Hurd P.D. (USNM); 5 km NE Huejucar, 1 ♀ 13/IX/1984, Pulawski W.J., 22.21 N 103.13 W (CAS); 5 mi. N Zacatecas, 1 ♀ 19/IX/1970, Bohart G.E. & R.M. Bohart (CNC); 9 mi. SE Fresnillo, 1 ♂ 7–14/VIII/1954, Linsley E.G., J.W. MacSwain & R.F. Smith (EMEC); Fresnillo, 1 ♂ 15/VIII/1947, 2134 m, Rockefeller D. (AMNH).

### Crassomicrodus clypealis

Figueroa, Sharkey & Romero sp. n.

urn:lsid:zoobank.org:act:1D3A68FB-656B-44F1-BC9E-B7E343BF4A90

http://species-id.net/wiki/Crassomicrodus_clypealis

Fig. 3a–e

#### Description female.

**Figure 3.**
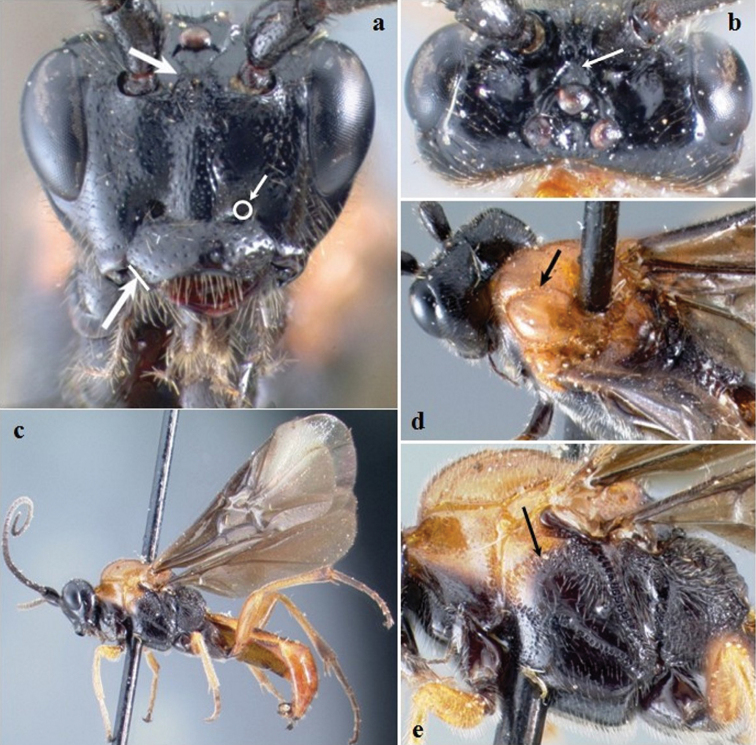
*Crassomicrodus clypealis* sp. n. Female **a** anterior view of head, arrows indicate a median pyramidal-shaped elevation with two weakly defined tubercles, ventrolateral margin of clypeus, and tentorial pit **b** dorsal view of head, arrow indicates frons deeply excavated with a pair of microfoveolate grooves **c** female habitus **d** dorsal view of mesosoma, arrows indicate impressed notauli **e** lateral view of mesosoma, arrow indicates subalar lobe separated from mesopleuron by narrow groove.

Body. Length. 7.38–7.50 mm. Color (Fig. 3c). Integument yellowish-orange except black as follows, mandible apex, head, antenna, propleuron, mesopleuron, metapleuron, metanotum, propodeum, coxa and trochanters; ocelli translucent honey yellow; apical area of hind tibia, middle and hind tarsomeres blackish; wing veins dark brown; forewing strongly infumate with a hyaline spot on the first submarginal cell that is similar in size to the parastigma. Head (Fig. 3ab). Transverse in frontal view; face with weak longitudinal ridge dorsomedially; eye height/width = 1.38–1.43; eye height 0.68–0.70× inter-ocular distance; area between antennal sockets with a median pyramidal-shaped elevation and two weakly defined tubercles; frons deeply excavated with a pair of microfoveolate grooves that diverge towards the ocellar area; posterior surface of antennal sockets smooth; groove between lateral ocelli smooth; median ocellus separated from lateral ocellus by smooth groove; gena slightly bulging; malar space 0.46–0.48× as long as eye height; clypeus 2.40–2.42× wider than high; length of ventrolateral margin of clypeus distinctly longer than diameter of tentorial pit; antenna with 38–39 flagellomeres; setae at base of mandible distinctly longer than setae on rest of body surface. Mesosoma (Fig. 3cde). Pronotum smooth; lateral pronotal margins with weakly crenulate groove; notauli impressed; anterolateral edges of scutellum lacking small acute projection; scutellar disc convex with sparse setae from 0.07 to 0.08 mm in length; scutellar disc sloped posteriorly and rounded; lateral scutellar depression with punctures and foveolae; carinae of central metanotal area in triangular shaped; propodeum reticulate rugose; subalar lobe separated from mesopleuron by narrow rugulose groove, width distinctly shorter than the subalar lobe; metapleuron reticulate rugulose or reticulate punctures. Legs. Inner spur of middle tibia 0.69–0.72× length of basitarsus; inner spur of hind tibia 0.55–0.58× length of basitarsus; metabasitarsus 1.15–1.23× length of tarsomeres III, IV, and V combined; hind tibia 2.25–2.30× longer than basitarsus; hind femur length 3.56–3.78× its maximum width. Wings. Forewing length/width = 2.42–2.50; stigma 2.88–3.00× longer than maximum width; forewing vein R1 0.58–0.62× as long as vein RS; vein RS sinuate; vein r arising slightly before middle of stigma; second submarginal cell triangular, with petiole 0.06–0.09 mm long; vein M+CU distinctly pigmented throughout; hind wing length/width = 2.91–3.15; hind wing vein 1M 1.35–1.43× longer than 1r-m; hind wing with 6–7 hamuli. Metasoma. Apical width of petiole (tergum 1) 2.82–3.14× wider than basal width; minimum width of petiole 0.56–0.64× apical width; length of ovipositor sheath 0.35–0.37 mm.

#### Male.

Similar to female, except antenna with 36 to 41 flagellomeres and pronotum may be slightly melanic.

#### Host.

Unkown.

#### Distribution.

USA.

#### Diagnosis.

Distinguished from other *Crassomicrodus* species by the following combination of characters: area between antennal sockets with a median pyramidal-shaped elevation, length of ventrolateral margin of clypeus distinctly longer than diameter of tentorial pit, and gena slightly bulging.

#### Remarks.

This species is near to male of *Crassomicrodus fulvescens*, but differs in that *Crassomicrodus fulvescens* has the length of ventrolateral margin of clypeus similar to the diameter of the tentorial pit; gena distinctly bulging; and forewing infumate with a large hyaline spot in first submarginal cell.

#### Etymology.

Named “clypealis” to emphasize that the ventral margin of the clypeus is longer than the diameter of each tentorial pit.

#### Material examined.

 Holotype ♀: USA, Colorado: nr. Roggen, 4/IX/1972, Lanham U.N. & C.C. Lanham. Allotype ♂: Roggen, 29/VIII/1930, Rodeck H.G.. Holotype and Allotype deposited in UCMC. Paratypes: 1 ♂; **USA, Colorado:** 3 mi. NW Roggen, Weld Co., 4/IX/1974, Lanham U.N. (CNC); 1 ♀ same data as holotype; 1 ♂ same data as allotype (UCMC); Sandhills N of Roggen Weld Co.: 1 ♂ 12/IX/1934, Rodeck H.G. (HIC); 1 ♂ 12/IX/1934, Rodeck H.G. (USNM); 2 ♂ 12/IX/1934, Rodeck H.G.; 1 ♂ 16/VIII/1990, Bowers M.D. (UCMC). **Kansas:** Ness Co., 1 ♂ 7/IX/1965, Blocker H.D. (KSUC). **Nebraska:** Halsey: 1 ♀, 1 ♂ 11/VIII/1925, 1 ♂ 12/VIII/1925, 1 ♂ 15/VIII/1925, Dawson R.W. (UMSP).

### Crassomicrodus costaricensis

Figueroa, Sharkey and Romero sp. n.

urn:lsid:zoobank.org:act:B9743BFC-4824-494E-BD02-B19BCBF66134

http://species-id.net/wiki/Crassomicrodus_costaricensis

Fig. 4a–e

#### Description female.

**Figure 4.**
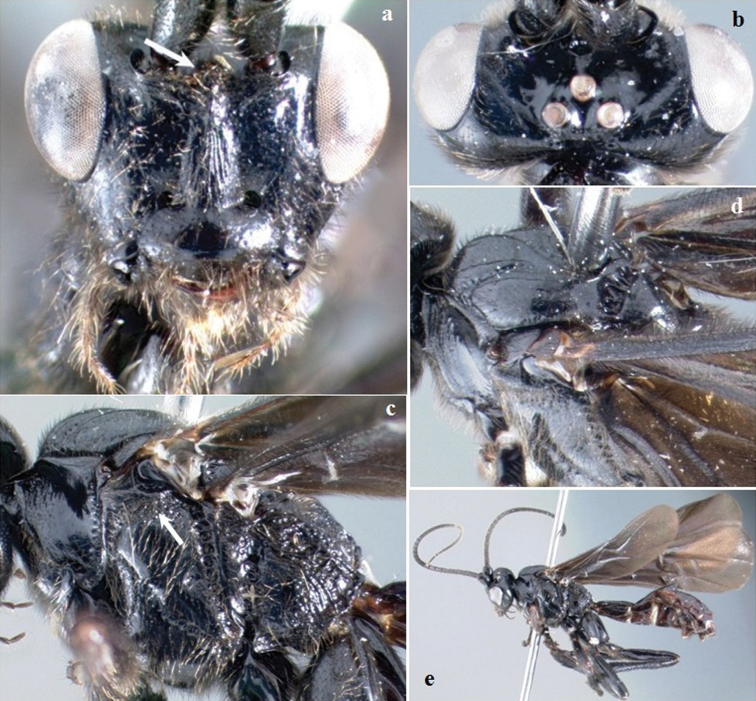
*Crassomicrodus costaricensis* sp. n. Female **a** anterior view of head, arrow indicates a median pyramidal-shaped elevation **b** dorsal view of head **c** lateral view of mesosoma, arrow indicates subalar lobe separated from mesopleuron by wide groove **d** dorsal view of mesosoma **e** female habitus.

Body. Length. 8.10–8.70 mm. Color (Fig. 4e). Integument black except eye silver, ocelli translucent yellow (Fig. 1d); medial area of mandible yellow-reddish; metasoma dark brown; forewing strongly infumate with a hyaline spot on the first submarginal cell that is similar in size to the parastigma. Head (Fig. 4ab). Triangular in frontal view; face with longitudinal ridge dorsomedially; eye height/width = 1.41–1.42; eye height (lateral view) 0.75–0.77× inter-ocular distance (anterior view); area between antennal sockets with a median pyramidal-shaped elevation; frons excavated with a little longitudinal groove; posterior surface of antennal sockets smooth; groove between lateral ocelli smooth; median ocellus separated from lateral ocellus by smooth groove; gena not bulging; malar space 0.47–0.51× as long as eye height; clypeus 2.13–2.32× wider than high; length of ventrolateral margin of clypeus similar to diameter of tentorial pit; antenna with 41 flagellomeres; setae at base of mandible distinctly longer than setae on rest of body surface. Mesosoma (Fig. 4cde). Pronotum punctulate with setae; lateral pronotal margins with a shallow, crenulate groove; notauli impressed; anterolateral edges of scutellum lacking small acute projection; scutellar disc convex with sparse setae from 0.12 to 0.14 mm in length; scutellar disc sloped posteriorly and rounded; lateral scutellar depression smooth with punctures or rugosities in its ventral border; carinae of central metanotal area almost circular shaped; propodeum reticulate rugulose, more pronounced on lateral margins; subalar lobe separated from mesopleuron by wide rugulose groove, width almost of similar size to subalar lobe; metapleuron smooth, only reticulate rugulose one-fourth of ventral area. Legs. Inner spur of middle tibia 0.76–0.89× length of basitarsus; inner spur of hind tibia 0.62–0.72× length of basitarsus; metabasitarsus 1.02–1.18× length of tarsomeres III, IV, and V combined; hind tibia 2.04–2.38× longer than basitarsus; hind femur length 4.07–4.17× its maximum width. Wings. Forewing length/width = 2.58; stigma 4.54–5.00× longer than maximum width; forewing vein R1 0.60–0.64× as long as vein RS; vein RS sinuate; vein r arising before middle of stigma; second submarginal cell triangular, with petiole 0.13–0.20 mm long; vein M+CU distinctly pigmented throughout; hind wing length/width = 3.48–3.65; hind wing vein 1M 1.01–1.08× longer than 1r-m; hind wing with 6–7 hamuli. Metasoma. Apical width of petiole (tergum 1) 2.86–2.91× wider than basal width; minimum width of petiole 0.49–0.59× apical width; length of ovipositor sheath 0.28–0.41 mm.

#### Male.

Unknown.

#### Host.

Unknown.

#### Distribution.

Costa Rica and El Salvador.

#### Diagnosis.

Distinguished from other *Crassomicrodus* species by the following combination of characters: area between antennal sockets with a median pyramidal-shaped elevation, eye height 0.75–0.77× inter-ocular distance, antenna with 41 flagellomeres, body length 8.10 to 8.70 mm, hind wing vein 1M 1.01–1.08× longer than 1r-m, head and mesosoma black, metasoma dark brown, and wings strongly infumate.

#### Remark.

 Specimens from Costa Rica are homogeneous in their measurements, but the specimen from El Salvador differs significantly. Nonetheless it is considered conspecific here due to similarity in other characters. More specimens and molecular data should easily test this hypothesis in the future.

#### Etymology.

 The specific name is a noun in apposition to *Crassomicrodus* and is chosen because of the locality where the holotype was collected, Costa Rica.

#### Material examined.

**Holotype** ♀**:** COSTA RICA, Guanacaste: Barra Honda NP, VI/1988, 200 m., Gauld & Mitchel, deposited in MUCR. Paratypes: 1 ♀ same data as holotype (MUCR); **Guanacaste:** Scrub forest (7yr), Open site, 1 ♀ 22/VI/1985, Gauld & Janzen, 300 m. (HIC). **EL SALVADOR, [La Unión]:** Vol. Conchagua, 1 ♀; 27–29/V/1958, Cartwright O.L. (MUCR).

### Crassomicrodus divisus

(Cresson, 1873)

http://species-id.net/wiki/Crassomicrodus_divisus

Fig. 5a–e

Crassomicrodus divisus (Cresson): Bradley 1916: 139–140; Muesebeck 1927: 21.
Microdus divisus Cresson 1873: 52 [Examined].
Orgilus rileyi Ashmead 1888: 640 [Examined].
Epimicrodus divisus Ashmead 1900: 129.

#### Holotype female.

 Illinois [USA]. No. 1726.1 (ANSP).

#### Description female.

**Figure 5.**
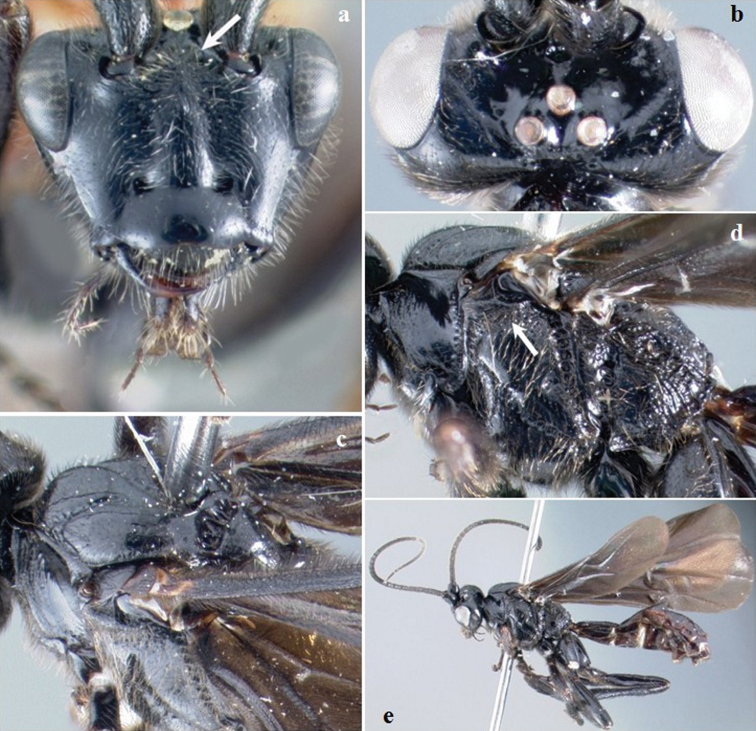
*Crassomicrodus divisus*. Female **a** anterior view of head, arrow indicates a median pyramidal-shaped elevation **b** dorsal view of head **c.** dorsal view of mesosoma **d** lateral view of mesosoma, arrow indicates subalar lobe separated from mesopleuron by wide groove **e.** female habitus.

 Body. Length. 6.95–8.60 mm. Color (Fig. 5e). Integument black except reddish yellow as follows, pronotum, mesonotum, subalar lobe, tegulae, hind femora, and metasoma; mandible and wing veins dark brown; eye silver or blackish; ocelli translucent yellow; forewing infumate with a hyaline spot on the first submarginal cell that is similar in size to the parastigma. Rarely, central area of mesopleuron or hind coxa or propodeum and metapleuron reddish yellow. Head (Fig. 5ab). Triangular in frontal view; face with longitudinal ridge dorsomedially; eye height/width = 1.38–1.40; eye height 0.57–0.58× inter-ocular distance; area between antennal sockets with a median pyramidal-shaped elevation, sometimes with two weakly defined tubercles; frons excavated with a pair of microfoveolate grooves that diverge towards the ocellar area; posterior surface of antennal sockets smooth; groove between lateral ocelli smooth; median ocellus separated from lateral ocellus by smooth groove; gena not bulging; malar space 0.78–0.83× as long as eye height; clypeus 1.85–1.95× wider than high; length of ventrolateral margin of clypeus similar to diameter of tentorial pit; antenna with 32–35 flagellomeres; setae at base of mandible distinctly longer than setae on rest of body surface. Mesosoma (Fig. 5cde). Pronotum reticulate rugulose, sometimes strigose; lateral pronotal margins with a shallow, crenulate groove; notauli impressed; anterolateral edges of scutellum lacking small acute projection; scutellar disc slightly convex with sparse setae from 0.10 to 0.11 mm in length; scutellar disc sloped posteriorly and flattened; lateral scutellar depression reticulate rugulose and foveolae; carinae of central metanotal area forming a triangular cell; propodeum reticulate rugose; subalar lobe separated from mesopleuron by wide rugose groove with reticulate foveolae, width almost of similar size to subalar lobe; metapleuron reticulate-rugose. Legs. Inner spur of middle tibia 0.67–0.71× length of basitarsus; inner spur of hind tibia 0.59–0.68× length of basitarsus; metabasitarsus 1.21–1.29× length of tarsomeres III, IV, and V combined; hind tibia 2.17–2.27× longer than basitarsus; hind femur length 3.91–4.22× its maximum width. Wings. Forewing length/width = 2.56–2.60; stigma 3.27–3.70× longer than maximum width; forewing vein R1 0.59–0.64× as long as vein RS; vein RS not sinuate; vein r arising before middle of stigma; second submarginal cell quadrangular, with petiole 0.12–0.21 mm long; vein M+CU distinctly pigmented throughout; hind wing length/width = 3.25–3.51; hind wing vein 1M 1.52–1.60× longer than 1r-m; hind wing with 6–7 hamuli. Metasoma. Apical width of petiole (tergum 1) 3.10–3.13× wider than basal width; minimum width of petiole 0.61–0.63× apical width; length of ovipositor sheath 0.33–0.41 mm.

#### Male.

Unknown.

#### Host.

Unknown.

#### Distribution.

Canada, Mexico, and USA.

#### Diagnosis.

Distinguished from other *Crassomicrodus* species by the following combination of characters: area between antennal sockets with a median pyramidal-shaped elevation, malar space 0.78–0.83× as long as eye height, scutellar disc sloped posteriorly and flattened, head black and mesosoma mostly black with some areas reddish yellow.

#### Remarks.

Males of *Crassomicrodus divisus* were not found in this revision, although Muesebeck (1927) recorded them. We carefully examined long series from diverse localities to find them but without success. Therefore we speculate that males of this species are absent or very rare.

#### Material examined.

 Type: *Microdus divisus* 1 ♀, Ill. (ANSP), *Orgilus riley* 1 ♀ (USNM). Homotype: 1 ♀, **Texas:** Davis Ranch, NW Blanco Co., 22/IV/1959, Mason W.R.M. (CNC).*Other specimens examined*.-1 ♀ (AMNH). 9 ♀ (INHS). 1 ♀(MSUC). 1 ♀ (USNM). 1 ♀; 19/VI/1898 (AEIC). 1 ♀; VII/1917 (USNM). 1 ♀; **CANADA, Ontario:** Constance Bay, Carleton Co., 24–31/VI/1977, Sanborne M. (CNC). 1 ♀;Ottawa, 3/X/1912, Beaulne J.I. (CNC). 1 ♀; Cana (USNM). 1 ♀; **MEXICO, Durango:** 10 miles N Durango, 12/VII/1954, McSwain (EMEC). 1 ♀; **Guanajuato:** San Miguel Allende, 12/VIII/1953, Vaurie C. & P. Vaurie (AMNH). 1 ♀; **Jalisco:** 4 miles W Mazamitla, 16/X/1950, 2073 m., Smith Ray F. (AMNH). 1 ♀;Guadalajara, 17/IX/1957, Dreisbach R. & K. Dreisbach (MSUC). 1 ♀;Guadalajara, 17–20/VII/1965, Evans H.E. (MCZ). 1 ♀; **Nuevo León:** 4 miles W El Cercado, 6/VI/1951, Hurd P.D. (EMEC). 1 ♀;9 miles W Iturbide, 3/VII/1974, Clark, Murray, Ashe, Schaffner (TAMU). 1 ♀; **Puebla:** Puebla, 3/VII/1952, Gilbert E.E. & C.D. MacNeil (EMEC). 1 ♀; **Zacatecas:** Trancosa, 3/VII/1961, Dreisbach R. & K. Dreisbach (MSUC). 4 ♀; **USA,** Colo. (USNM). 1 ♀;Detroit, 7/VI (USNM). 1 ♀; **Arizona:** Canelo, 10/VII/1957, Butler G.D. (HIC). 1 ♀; **Illinois:** Algonquin, 30/VIII/94–99 (INHS). 1 ♀;Algonquin, Nason (INHS). 1 ♀;Grand Tower, 28/VII/1905 (INHS). 1 ♀;Illinois, VIII/1899 (MCZ). 1 ♀;Meredosia, 20/VIII/1917 (INHS). 1 ♀; **Iowa:** Ames, 25/V/1925 (UCDC). 1 ♀;Sioux City, Ainslie C.N. (USNM). 1 ♀; **Kansas:** Baldwin, V/190?, Bridwell J.C. (USNM). 1 ♀;Baldwin, VIII, Bridwell J.C. (UCDC). 1 ♀;Douglas Co. (MCZ). 1 ♀;Manhattan, 10/VI/1950, Evans H.E. (KSUC). 1 ♀;Manhattan, 10/VII/1949, Kring James B. (KSUC). 1 ♀;Onaga, 9/VII/1922, Crevecoeur (KSUC). 1 ♀;Riley Co., 16/VI, Smith R.C. (KSUC). 1 ♀;Riley Co., 9/VII, Popenoe (KSUC). 1 ♀;Riley Co., V, Marlatt (KSUC). 1 ♀; **Kentucky:** Lexington (USNM). 1 ♀; **Michigan:** White Pigeon, St. Joseph Co., 14/VI/1959, Fischer R.L. (MSUC). 1 ♀; **Minnesota:** Lake Pepin E Frontenac, 24/V/1941, Sun V.P. (UMSP). 1 ♀;Ortonville, 5/VIII/1935, Denning D.G. (UMSP). 1 ♀;Olmsted Co.,VIII/1896, Linslie C.N.(USNM).1 ♀; **Missouri:** Columbia, Boone Co., 16/VIII/1968, Parker F.D., malaise trap (HIC). 1 ♀;Clayton, 3/VI/1939, Pickel B.H. (AEIC). 1 ♀; Columbia, 1/VIII/1968, Parker F.D. malaise trap (UCDC). 1 ♀;Columbia, 10/VIII/1967, Parker F.D. malaise trap (USNM). 1 ♀;Columbia, 14/IX/1939, Crajo W.S. (UMRM). 1 ♀;Columbia, 17/VIII/1966, Huber S.F. (USNM). 1 ♀;Columbia, 2/VIII/1968, Parker F.D. malaise trap (UCDC). 1 ♀;Columbia, 23/VIII/1967, Parker F.D. malaise trap (USNM). 2 ♀;Columbia, 26/VIII/1967, Parker F.D. malaise trap (USNM). 1 ♀;Columbia, 10/VII/1967, Parker F.D., malaise trap (USNM). 1 ♀;Columbia, 22/VII/1967, Parker F.D., malaise trap (USNM). 1 ♀;Columbia, 20/IX/1967, Parker F.D., malaise trap (USNM). 1 ♀;Columbia, 6/IX/1967, Parker F.D. malaise trap (USNM). 1 ♀;Columbia, 7/IX/1966, Huber S.F. (USNM). 1 ♀;Columbia, 16/VIII/1966, Huber S.F. (USNM). 2 ♀;Columbia, Boone Co., 22/VIII/1968, Parker F.D. malaise trap (USNM). 2 ♀;Jefferson City, 6/VII/1941, Adams C.F. (USNM). 1 ♀;Sapp, 10/VII/1954 (UMRM). 1 ♀; **Nebraska:** Maxwell, 25/VII/1967 (IRCW). 1 ♀; **New Jersey:** Camden, 1892? (MSUC). 1 ♀;Trenton, Abbatt (MCZ). 1 ♀; **New York:** Boston, 1/VIII/1909, M.C.V. (UCDC). 1 ♀;Lancaster, 10/VIII/1891, E.P.V. (CAS). 1 ♀; **Ohio:** Champaign Co., 23/VII/1941, Guillaspy J.F. (OSU). 1 ♀;Champaign Co., 23/VII/1941, Guillaspy J.F. (OSU). 1 ♀;Dayton O., VIII/1927, Basker C.A. (OSU). 1 ♀;W Jefferson, Franklin Co. (USNM). 1 ♀; **South** **Dakota:** Vermillion, 30/VI/1960, Walgenbach D.D. (IRCW). 1 ♀;Davis Ranch, NW Blanco Co., 22/IV/1959, Mason W.R.M. (CNC). 1 ♀;Edna, 19/VII/1908, Mitchell J.D. (USNM). 1 ♀; **Texas:** Sonora, Sutton Co., 19/V/1973, Menke & Miller (USNM). 1 ♀; **Virginia?:** Va. (USNM). 1 ♀; **Wisconsin:** Columbia Co., 23/VII/1961, Carney Don (IRCW). 1 ♀; Dane Co., VIII/1899 (AEIC). 1 ♀; Dane Co., 10/VIII/1899 (AEIC). 1 ♀; St. Croix Co., 2/VIII/1916, McNeel W. (AEIC).

### Crassomicrodus fulvescens

(Cresson, 1865)

http://species-id.net/wiki/Crassomicrodus_fulvescens

Microdus fulvescens Cresson 1865: 297 [Examined].
Microdus medius Cresson 1865: 298 [Examined].

#### Holotype female.

 Col. No. 1727.1 (ANSP).

This species was recently investigated by Figueroa et al. (2008), who found that *Crassomicrodus medius*, based on males only, is conspecific with *Crassomicrodus fulvescens*, which was based entirely on females.

### Crassomicrodus jalisciensis

Figueroa, Romero & Sharkey sp. n.

urn:lsid:zoobank.org:act:E141B22C-E1E6-458A-A817-9550A6ED2FDA

http://species-id.net/wiki/Crassomicrodus_jalisciensis

Fig. 6a–e

#### Description female.

**Figure 6.**
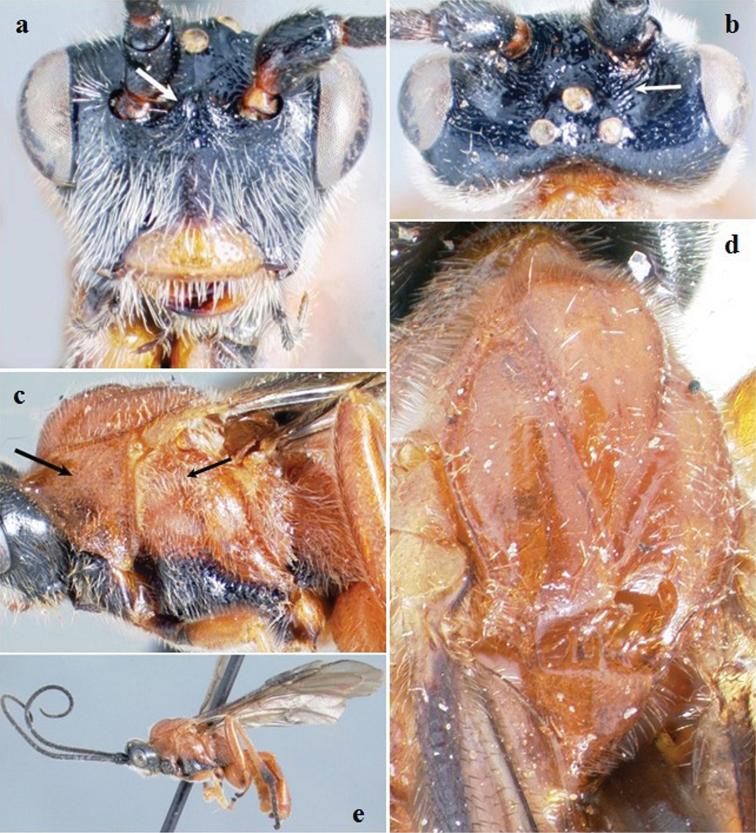
*Crassomicrodus jalisciensis*. Female **a** anterior view of head, arrow indicates a median pyramidal-shaped elevation **b** dorsal view of head, arrow indicates posterior surface of antennal sockets rugulose **c** lateral view of mesosoma, arrows indicate pronotum and subalar lobe separated from mesopleuron by wide groove **d** dorsal view of mesosoma **e** female habitus.

Body. Length. 7.35–7.50 mm. Color (Fig. 6e). Integument yellowish orange except black as follows, face, frons, gena temple, vertex, antenna, mandible apex, propleuron, ventral area of mesopleuron, apical area of hind tibia and tarsomeres; eye silver or blackish, ocelli translucent yellow; blackish; wing veins dark brown; forewing infumate with a hyaline spot on the first submarginal cell that is similar in size to the parastigma. Sometimes trochanters blackish and/or propleuron yellowish orange. Head (Fig. 6ab). Triangular in frontal view; face without longitudinal ridge dorsomedially; eye height/width = 1.34–1.45; eye height 0.59–0.61× inter-ocular distance; area between antennal sockets with a median pyramidal-shaped elevation; frons deeply excavated and crenulate with a pair of microfoveolate grooves that diverge towards the ocellar area; posterior surface of antennal sockets rugulose; groove between lateral ocelli smooth; median ocellus separated from lateral ocellus by smooth groove; gena not bulging; malar space 0.58–0.63× as long as eye height; clypeus 2.40–2.50× wider than high; length of ventrolateral margin of clypeus similar to diameter of tentorial pit; antenna with 38–40 flagellomeres; setae at base of mandible slightly longer than setae on rest of body surface; face setose. Mesosoma (Fig. 6cde). Pronotum strigose; lateral pronotal margins with weakly crenulate groove; notauli impressed; anterolateral edges of scutellum lacking small acute projection; scutellar disc convex with sparse setae from 0.13 to 0.15 mm in length; scutellar disc sloped posteriorly and rounded; lateral scutellar depression punctulate; carinae of central metanotal area almost pentagonal shaped with the top inverted; propodeum reticulate rugulose; subalar lobe separated from mesopleuron by wide rugulose groove, width almost of similar size to subalar lobe; metapleuron reticulate-rugulose. Legs. Inner spur of middle tibia 0.69–0.78× length of basitarsus; inner spur of hind tibia 0.61–0.78× length of basitarsus; metabasitarsus 1.24–1.26× length of tarsomeres III, IV, and V combined; hind tibia 2.50–2.63× longer than basitarsus; hind femur length 4.54–4.76× its maximum width. Wings. Forewing length/width = 2.72–3.02; stigma 3.45–3.57× longer than maximum width; forewing vein R1 0.63–0.69× as long as vein RS; vein RS sinuate; vein r arising before middle of stigma; second submarginal cell triangular, with petiole 0.09–0.11 mm long; vein M+CU distinctly pigmented throughout; hind wing length/width = 3.76–4.11; hind wing vein 1M 1.56–1.64× longer than 1r-m; hind wing with 5–6 hamuli. Metasoma. Apical width of petiole (tergum 1) 3.07–3.23× wider than basal width; minimum width of petiole 0.54–0.58× apical width; length of ovipositor sheath 0.30–0.33 mm.

#### Male.

Similar to female except color as follows: head, propleuron, pronotum, scutellum, metanotum, propodeum, mesopleuron, subalar lobe, metapleuron, coxae and trochanters black; inner spur of middle tibia almost half length of basitarsus (0.55×).

#### Host.

Unknown

#### Distribution.

Mexico.

#### Diagnosis.

Distinguished from other *Crassomicrodus* species by the following combination of characters: area between antennal sockets with a median pyramidal-shaped elevation, posterior surface of antennal sockets rugulose, face setose, setae at base of mandible slightly longer than setae on rest of body surface, subalar lobe separated from mesopleuron by wide rugulose groove, and mesosoma mostly yellowish orange with wings infumate.

#### Remark.

This speciesis near *Crassomicrodus oaxaquensis*, but differs in that *Crassomicrodus oaxaquensis* has the mesosoma black; wings hyaline; face with a weak longitudinal ridge dorsomedially; area between antennal sockets with a median pyramidal-shaped elevation and two weakly defined tubercles. One specimen of *Crassomicrodus jalisciensis* has the head and mesosoma black, but differs from *Crassomicrodus oaxaquensis* by leg and wing coloration.

#### Etymology.

*Crassomicrodus jalisciensis* refers to the state of Jalisco, where all specimens have been found.

#### Material examined.

 Holotype ♀: **MEXICO, Jalisco:** 9 miles W Tepatitlán, El Refugio, 3/VII/1953, C. Vaurie & P. Vaurie. Allotype ♂: same data as holotype. Paratypes: 2 ♀ same data as holotype; Guadalajara, 1 ♀ 23–28/VII/1965, H.E. Evans (MCZ); 8 miles S Guadalajara, 1 ♀ 10/VII/1963, Parker F.D. & L.A. Stange (USNM); Guadalajara, 2 ♂ 16/VII/1951, 2 ♂ 1 ♀ 17/VII/1951, Evans H.E. (AEIC). Holotype and allotype and paratypes with same data deposited in AMNH.

### Crassomicrodus mariae

Figueroa, Sharkey & Romero sp. n.

urn:lsid:zoobank.org:act:D958480B-3DC0-4377-95BB-AB49F0B381F8

http://species-id.net/wiki/Crassomicrodus_mariae

Fig. 7a–e

#### Description female. 

**Figure 7.**
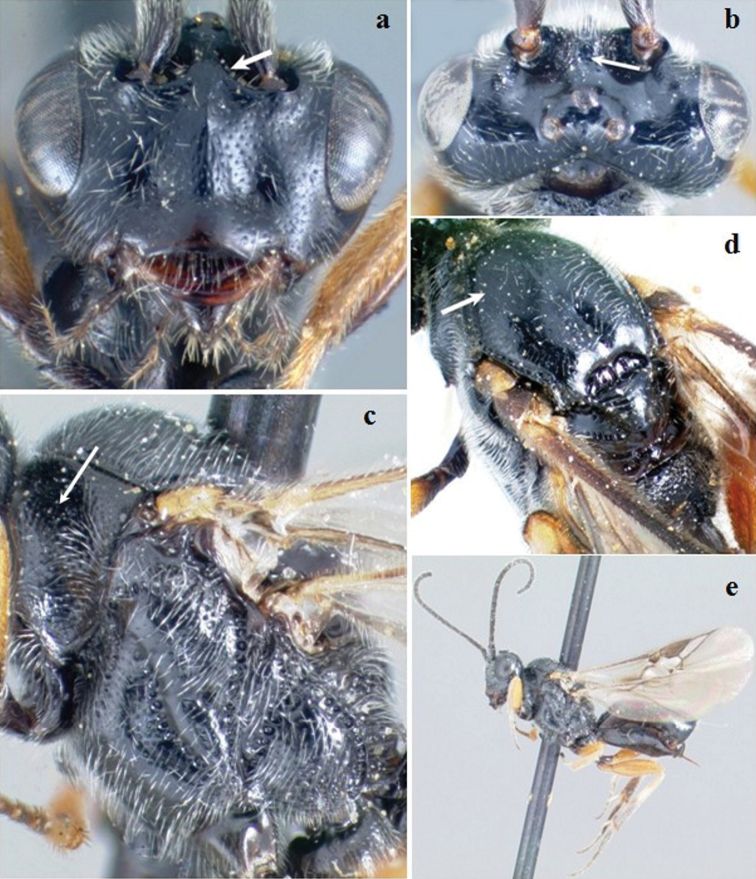
*Crassomicrodus mariae*. Female **a** anterior view of head, arrow indicates a median pyramidal-shaped elevation **b** dorsal view of head, arrow indicates frons not excavated **c** lateral view of mesosoma, arrow indicates punctate pronotum **d** dorsal view of mesosoma, arrow indicates notauli not impressed **e** female habitus.

Body. Length. 5.13–5.38 mm. Color (Fig. 7e). Integument black except yellowish-orange as follows, basal area of mandible, tegulae, femora, fore and middle tibia; medial area of hind tibia pale yellow; ocelli translucent yellow; apical area of mandible reddish; tarsomeres and apical area of hind tibia blackish; tergum dark brown; sternum and wing veins brown; forewing lightly infumate with a hyaline spot on the first submarginal cell that is similar in size to the parastigma. Head (Fig. 7ab). Transverse in frontal view; face with longitudinal ridge dorsomedially; eye height/width = 1.35–1.42; eye height 0.67–0.72× inter-ocular distance; area between antennal sockets with a median pyramidal-shaped elevation; frons not excavated; posterior surface of antennal sockets smooth; groove between lateral ocelli smooth; median ocellus separated from lateral ocellus by smooth groove; gena bulging; malar space 0.38–0.43× as long as eye height; clypeus 2.53–2.67× wider than high; length of ventrolateral margin of clypeus distinctly longer than diameter of tentorial pit; antenna with 25 flagellomeres; setae at base of mandible distinctly longer than setae on rest of body surface. Mesosoma (Fig. 1cde). Pronotum punctuate; lateral pronotal margins with weakly crenulate groove; notauli not impressed; anterolateral edges of scutellum lacking small acute projection; scutellar disc convex with sparse setae from 0.09 to 0.10 mm in length; scutellar disc sloped posteriorly and rounded; lateral scutellar depression smooth centrally and microfoveolate on the margins; carinae of central metanotal area almost circular shaped; propodeum reticulate rugulose, more pronounced on lateral margins; subalar lobe separated from mesopleuron by narrow rugulose groove, width distinctly shorter than the subalar lobe; ventral one-fourth of metapleuron reticulate punctuate, remainder smooth. Legs. Inner spur of middle tibia 0.67–0.74× length of basitarsus; inner spur of hind tibia 0.52–0.53× length of basitarsus; metabasitarsus 1.35–1.42× length of tarsomeres III, IV, and V combined; hind tibia 2.21–2.30× longer than basitarsus; hind femur length 3.14–3.23× its maximum width. Wings. Forewing length/width = 2.07–2.17; stigma 2.65–2.75× longer than maximum width; forewing vein R1 0.48–0.50× as long as vein RS; vein RS slightly sinuate; vein r arising at middle of stigma; second submarginal cell triangular, with petiole 0.03–0.06 mm long; vein M+CU not pigmented throughout; hind wing length/width = 2.96–3.30; hind wing vein 1M 2.00–2.35× longer than 1r-m; hind wing with 4 hamuli. Metasoma. Apical width of petiole (tergum 1) 2.17–2.38× wider than basal width; minimum width of petiole 0.70–0.77× apical width; length of ovipositor sheath 0.76–0.78 mm.

#### Male.

Similar to female except male has 26–28 flagellomeres, carinae of central metanotal area almost pentagonal shaped, 4–5 hamuli.

#### Host.

Unknown

#### Distribution.

USA.

#### Diagnosis.

Distinguished from other *Crassomicrodus* species by the following combination of characters: area between antennal sockets with a median pyramidal-shaped elevation, frons not excavated, antenna with 25 flagellomeres, notauli not impressed, length of ovipositor sheath 0.76–0.78 mm, forewing vein R1 0.48–0.50× as long as vein RS, head and mesosoma black, and wings lightly infumate.

#### Remarks.

This species is near to *Crassomicrodus muesebecki*, but differs in that *Crassomicrodus muesebecki* measures 6.08 a 6.95 mm, area between antennal sockets with a median transverse elevation and two weakly defined lateral tubercles, frons deeply excavated, antenna with 28–29 flagellomeres, pronotum more smooth, sparse setae on scutellar disc from 0.18 to 0.20 mm in length, length of ovipositor sheath 1.83–2.33 mm, and coloration of metasoma is black.

#### Etymology.

 This species is named in honor of María Espinosa Morales, wife of the first author.

#### Material examined.

 Holotype ♀: USA, California: 5 miles W Llano, 2/V/1937, Timberlake, deposited in USNM. Allotype ♂: USA, Nevada: Patrick, Washoe Co., 22/VI/1971, Bohart R.M., deposited in UCDC. Paratypes: USA, **California:** Apple Valley, 1 ♂ 20/V/1955, Mason W.R.M. (CNC); Colton, 1 ♀ Eddy F.A. (USNM); Hwy 76 at junc. to Mt. Palomar, San Diego Co., 1 ♂ 26/VI/1976, Coville R.E. (EMEC); Mojave Desert Love Joy Butte, 1 ♀ 10/V/1944, Melander A.L. (UCR); Sagehen Creek nr. Hobart Mills, Nevada Co., 1 ♂ 24/VI/1964, Froebe J.A. (UCDC). **Colorado:** Limon, 1 ♂ 16/VIII/1949, Dreisbach R.R. & R.K. Schwab (USNM). **Nevada:** 15 miles E Reno, Nevada Co., 1 ♂ 4/VI/1963, Irwin M.E.; 4 ♂ same data as Allotype; 1 ♂ same data as Allotype but collected by Grissell E.E. (UCDC). 2 miles Nixon Washoe Co., 1 ♀ 22/VI/1961, Parker F.D.; 12 miles NE Stillwater Churchill, 2 ♀ 3/VI/1961, Parker F.D.; Winnemucca, Humboldt Co., 1 ♀ 15/VI/1960, Parker F.D.; Winnemucca, 1 ♀ 30/V/960, Haig T.R. (USNM). **Utah:** Wild Horse Cr., N Goblin Valley, Emery Co., 1 ♀ 3/VI/1982, 1494 m, Parker F.D. & Griswold T. (CNC).

### Crassomicrodus melanopleurus

(Ashmead, 1894)

http://species-id.net/wiki/Crassomicrodus_melanopleurus

Fig. 8a–e

Microdus melanopleurus Ashmead 1894: 125 [Examined]. Syn. n.

#### Holotype male.

 San Jose del Cabo. Cat. No. 223 (CAS)

#### Description female.

**Figure 8.**
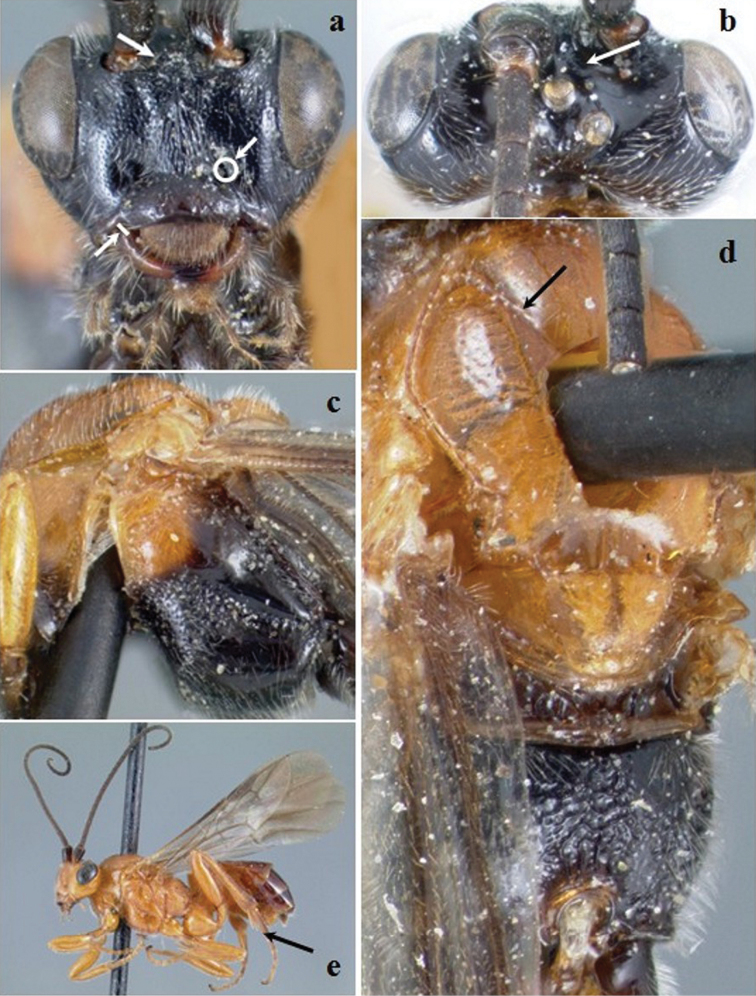
*Crassomicrodus melanopleurus*. Female **a** anterior view of head, arrows indicate a median pyramidal-shaped elevation with two weakly defined tubercles ventrolateral margin of clypeus, and tentorial pit **b** dorsal view of head, arrow indicates frons excavated with a pair of microfoveolate grooves **c** lateral view of mesosoma **d** dorsal view of mesosoma, arrow indicates impressed notauli **e** female habitus, arrow indicates inner spur of hind tibia.

 Body. Length. 6.10–7.75 mm. Color (Fig. 8e).Integument yellowish orange except ocelli translucent yellow reddish; antenna brown or black; eye, apical area of mandible and sometimes apical area of hind tibia and tarsomeres blackish; sometimes head and propleuron black; wing veins dark brown; forewing infumate with a hyaline spot on the first submarginal cell that is bigger than parastigma, sometimes forewing slightly infumate without distinguished the hyaline spot. Head (Fig. 8ab)**.** Transverse in frontal view; face without longitudinal ridge dorsomedially; eye height/width = 1.38–1.39; eye height 0.67–0.69× inter-ocular distance; area between antennal sockets with a median pyramidal-shaped elevation and two weakly defined tubercles; frons excavated with a pair of microfoveolate grooves that diverge towards the ocellar area; posterior area of antennal sockets smooth; groove between lateral ocelli smooth; median ocellus separated from lateral ocellus by smooth groove; gena bulging; malar space 0.48–0.57× as long as eye height; clypeus 2.40–2.55× wider than high; length of ventrolateral margin of clypeus similar to diameter of tentorial pit; antenna with 35–38 flagellomeres; setae at base of mandible distinctly longer than setae on rest of body surface. Mesosoma (Fig. 8cde)**.** Pronotum smooth; lateral pronotal margins with weakly crenulate groove; notauli impressed; anterolateral edges of scutellum lacking small acute projection; scutellar disc convex with sparse setae from 0.09 to 0.11 mm in length; scutellar disc sloped posteriorly and rounded; lateral scutellar depression smooth with punctures on the ventral margin; carinae of central metanotal area forming a triangular cell; propodeum reticulate rugulose, sometimes rugose; subalar lobe separated from mesopleuron by narrow rugulose groove, width distinctly shorter than the subalar lobe; ventral three-fourths of metapleuron reticulate rugulose, remainder with punctures. Legs. Inner spur of middle tibia 0.77–0.86× length of basitarsus; inner spur of hind tibia 0.60–0.72× length of basitarsus; metabasitarsus 1.11–1.19× length of tarsomeres III, IV, and V combined; hind tibia 2.17–2.38× longer than basitarsus; hind femur length 3.50–3.85× its maximum width. Wings. Forewing length/width = 2.72–2.76; stigma 3.43–3.55× longer than maximum width; forewing vein R1 0.61–0.67× as long as vein RS; vein RS not sinuate; vein r arising before middle of stigma; second submarginal cell triangular, with petiole 0.07–0.15 mm long; vein M+CU distinctly pigmented throughout; hind wing length/width = 3.60–4.10; hind wing vein 1M 1.55–1.85× longer than 1r-m; hind wing with 5–8 hamuli. Metasoma. Apical width of petiole (tergum 1) 3.00–3.70× wider than basal width; minimum width of petiole 0.54–0.56× apical width; length of ovipositor sheath 0.20–0.33 mm.

#### Male.

Similar to female except color as follows: head, propleuron, mesopleuron, metapleuron, propodeum, coxa and trochanters black; sometimes coloration similar to female.

#### Host.

Unknown.

#### Distribution.

Mexico and USA.

#### Diagnosis.

Distinguished from other *Crassomicrodus* species by the following combination of characters: area between antennal sockets with a median pyramidal-shaped elevation, head transverse in frontal view, gena bulging, inner spur of middle tibia 0.77–0.86× length of basitarsus; inner spur of hind tibia 0.60–0.72× length of basitarsus, and body mostly yellowish orange with wings infumate.

#### Remarks.

This species is difficult to circumscribe, the head shape and general coloration have a wide range of variation. We found some specimens with the head triangular in frontal view but they have the forewing infumate with a hyaline spot on the first submarginal cell, that occupies most of the space of the cell. Muesebeck (1927) suggested that the species could represent males of *Crassomicrodus fulvescens*. However the type of *Microdus melanopleurus* does not correspond to the characters of *Crassomicrodus fulvescens*, therefore we consider it to be a valid species. *Crassomicrodus melanopleurus* is very similar to *Crassomicrodus fulvescens*, but differs in that *Crassomicrodus fulvescens* has the gena distinctly bulging; frons deeply excavated; lateral scutellar depression rugose and foveolate; carinae of central metanotal defining an almost circular cell; inner spur of middle tibia 0.54–0.58× length of basitarsus; inner spur of hind tibia 0.48–0.54× length of basitarsus; and metapleuron completely reticulate-rugose.

#### Material examined.

Holotype ♂: MEXICO, San José del Cabo (CAS). *Other specimens examined*.- MEXICO, San Luis Potosí: K398, 25 miles N Tamazuncha, 1 ♂ 21/VIII/1960, Howden H. (CNC). USA, California: 15 miles E Baker, Cronese Wash, San Bernardino Co., 1 ♀ 3 ♂ 17/IV/1981, Pulawski W.J. (CAS); 15 miles W Baker, San Bernardino Co., 7 ♀ y 7 ♂ 17/IV/1981, Bohart R.M. (UCDC). Banning, Riverside Co., 2 ♂ 2/VII/1952, Grigarick A.A.; 1 ♂ 2/VII/1952, Mathis H.L.; 2 ♀ 27/VI/1952, Evans E.M.; 1 ♀ 27/VII/1952, Barcus D.E.; 1 ♀ 27/VI/1952, Miyagawa S.; 1 ♂ 28/VI/1952, Miyagawa S. (UCDC); 1 ♂ 28/VI/1952, Nakata J.H.; 1 ♂ 16/VII/1950, Adelson B. (USNM). Cronese Valley, San Bernardino Co., 1 ♂ 3/IV/1953, MacSwain J.W. (EMEC); 3 ♀ 4 ♂ 25/IV/1978, Smith N.J. (UCDC); 1 ♂ 25/IV/1978, Smith N.J. (USNM). Del Puerto Cyn, Stanislaus Co., 1 ♂ 12/VI/1978, Bohart R.M. (UCDC). Palm Springs Sta. Riverside Co., 5 ♂ 22/VII/1952, Menn J.J.; 1 ♀ y 3 ♂ 21/VII/1952, MacSwain J.W.; 1 ♂ 6/VII/1975, Linsley E.G. & J.M. Linsley (EMEC); 1 ♂ 22/VII/1952, Menn J.J. (HIC); 1 ♂ 19/V/1941, Knull D.J. & J.N. Knull (OSU); 4 ♀ 22/VII/1952, Barcus D.E. (UCDC); 1 ♀ y 2 ♂ 22/VII/1952, Menn J.J.; 1 ♂ 21/VII/1952, Thompson D.S.; 1 ♂ 22/VII/1952, Barcus D.E. (USNM). Tanbark Flat, Los Angeles Co., 2 ♀ 26/VI/1952, Anderson R.L.; Tanbark Flat, Los Angeles Co., 1 ♂ 12/VII/1952, Grigarick A.A. (UCDC); 1 ♂ 23/VII/1952, Evans E.M. (USNM). Tracy, San Joaquin Co., 1 ♂ 27/VII/1950, MacSwain J.W. (EMEC).

### Crassomicrodus muesebecki

Marsh, 1960

http://species-id.net/wiki/Crassomicrodus_muesebecki

Fig. 9a–e

Crassomicrodus muesebecki Marsh 1960: 153–154 [Examined].

#### Holotype female.

 7 miles Southwest of Trimmer, Fresno County, California [USA]. June 2, 1951. Cat. No. 64876 (USNM)

#### Description female.

**Figure 9.**
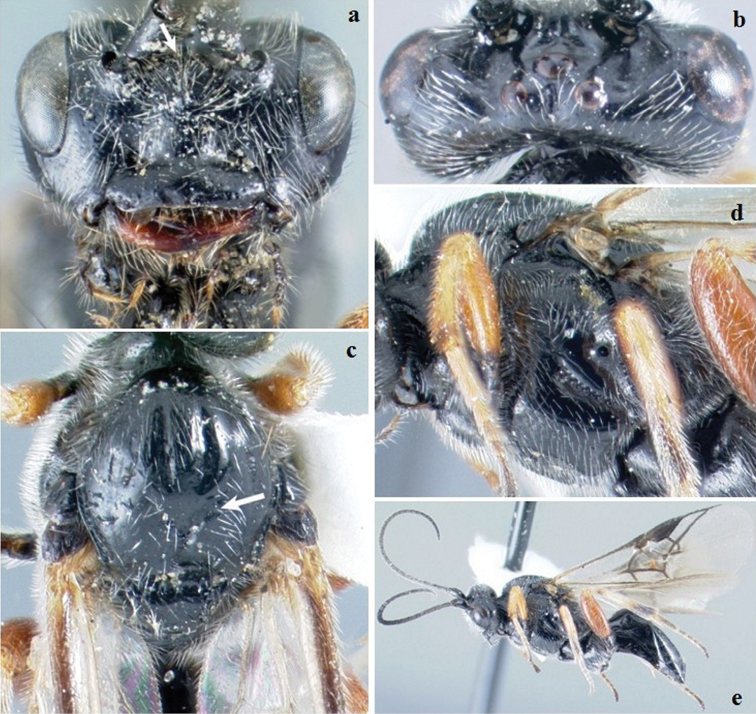
*Crassomicrodus muesebecki*. Female **a** anterior view of head, arrows indicate a median transverse elevation with two weakly defined tubercles **b** dorsal view of head **c** dorsal view of mesosoma, arrow indicates notauli not impressed **d** lateral view of mesosoma **e** female habitus.

Body. Length. 6.08–6.95 mm. Color (Fig. 9e). Integument black except yellowish orange as follows, femora, one-fourth basal area of hind tibia, fore and middle tibia with its tarsomeres; medial areas of mandible yellow reddish; eyes silver or blackish; ocelli translucent yellow; medial area of hind tibia pale yellow, apical area of hind tibia with its tarsomeres blackish; wing veins dark brown; forewing slightly infumate with a hyaline spot on the first submarginal cell that is similar in size to the parastigma. Head (Fig. 9ab). Transverse in frontal view; face with weak longitudinal ridge dorsomedially; eye height/width = 1.41–1.45; eye height 0.61–0.62× inter-ocular distance; area between antennal sockets with a median transverse elevation and two weakly defined lateral tubercles, sometimes tubercles not defined; frons deeply excavated with two or more foveolae on center, posteriorly continue a pair of smooth groove that diverge towards the ocellar area, sometimes microfoveolate; posterior surface of antennal sockets smooth; groove between lateral ocelli smooth; median ocellus separated from lateral ocellus by smooth groove; gena distinctly bulging; malar space 0.46–0.50× as long as eye height; clypeus 2.30–2.50× wider than high; length of ventrolateral margin of clypeus similar to diameter of tentorial pit; antenna with 28–29 flagellomeres; setae at base of mandible similar on size than setae on rest of body surface. Mesosoma (Fig. 9cde). Pronotum smooth with abundant setae on pronotal groove and lateral pronotal area; lateral pronotal margins with weakly crenulate groove; notauli not impressed; anterolateral edges of scutellum lacking small acute projection; scutellar disc convex with sparse setae from 0.18 to 0.20 mm in length; scutellar disc sloped posteriorly and rounded; lateral scutellar depression microfoveolate centrally, with rugosities and foveolae on the margins; carinae of central metanotal area almost circular shaped; propodeum reticulate rugulose, more pronounced on lateral margins; subalar lobe separated from mesopleuron by narrow rugose reticulate groove, width distinctly shorter than the subalar lobe; three-fourth dorsal area of metapleuron smooth, rest reticulate-punctuate. Legs. Inner spur of middle tibia 0.72–0.86× length of basitarsus; inner spur of hind tibia 0.59–0.67× length of basitarsus; metabasitarsus 1.03–1.18× length of tarsomeres III, IV, and V combined; hind tibia 2.63–2.81× longer than basitarsus; hind femur length 3.09–3.33× its maximum width. Wings. Forewing length/width = 2.51–2.53; stigma 2.62–3.00× longer than maximum width; forewing vein R1 0.59–0.64× as long as vein RS; vein RS not sinuate; vein r arising at middle of stigma; second submarginal cell triangular, with petiole 0.04–0.11 mm long; vein M+CU not pigmented throughout; hind wing length/width = 3.26–3.44; hind wing vein 1M 1.66–1.76× longer than 1r-m; hind wing with 4–5 hamuli. Metasoma. Apical width of petiole (tergum 1) 1.78–2.11× wider than basal width; minimum width of petiole 0.67–0.70× apical width; length of ovipositor sheath 1.83–2.33 mm.

#### Male.

Similar to female.

#### Host.

Unknown.

#### Distribution.

USA.

#### Diagnosis.

 Distinguished from other *Crassomicrodus* species by the following combination of characters: area between antennal sockets with a median transverse elevation, gena distinctly bulging, setae at base of mandible similar on size than setae on rest of body surface, notauli not impressed, scutellar disc convex with sparse setae from 0.18 to 0.20 mm in length, length of ovipositor sheath 1.83–2.33 mm, and body black with wings slightly infumate.

#### Remarks.

*Crassomicrodus muesebecki* Marsh was described with observations on 14 specimens. In this revision, we included 13 of these, one homotype and 14 additional specimens. With the inclusion of these new specimens we confirm Marsh’s (1960) observation that specimens from central and northern California have infumate wings, and in almost all the hind tibiae are yellowish orange, whereas the more southern specimens have hyaline wings and the medial areas of hind tibiae pale yellow. The species status of these two groups is tentative.

#### Material examined.

 Holotype ♀: **USA, California:** 7 mi. SW Trimmer, Fresco Co. 2/VI/1951, Mac Neill C.D. (USNM). Paratypes revised: **USA,** **California:** Keen Camp, San Jacinto Mts., 2 ♀ 31/V/1939, Bush W.C. (EMEC); 1 ♀ 31/V/1939, Smith Ray F. (USNM). Ribbonwood, San Jacinto Mts., 1 ♀ 21/V/1940, Michener C.D. (EMEC); 1 ♀ 21/V/1940, Michener C.D. (USNM). Tuolumne County, 1 ♀ 9/VI/1938, 1067 m., Hardman N.W. (EMEC); 1 ♀ 9/VI/1938, 1067 m., Hardman N.W. (USNM). Bucks Lake, Plumas County, 1 ♀ 23/VI/1949, Cox D. (CAS). Bass Lake, Madera County, 1 ♀ 6/VI/1938, Bohart R.M.; Rucker Lake, Nevada Co., 1 ♀ 5/VII/1949, Schlinger E.I.; Rumsey, Yolo Co., 1 ♀ 30/V/1956, Bohart R.M. (UCDC). Idyllwild, San Jacinto Mountains, 1 ♂ 19/VI/1951, Bechtel G.C. (USNM). Homotype ♀: Pinon Flat, San Jacinto Mts., 15/V/1941, Van Dyke E.C. (CNC). *Other specimens examined*.- **California**: Genoa, 3 ♀ 2 ♂ 26/VI/1948, Townes H.M.G. & D. Townes (AEIC); Keen Camp, 2 miles W San Jacinto Mts., 1 ♀ 31/V/1939, Laningham T.E.; Tuolumne County, 1 ♂9/VI/1938, 1067 m. (EMEC); Ribbonwood, San Jacinto Mountains, 1 ♀ 21/V/1940, Michener C.D. (HIC); Kelso Dunes, MNP, San Bernardino Co., 1 ♂19/V/2001, 770 m., Hawks D. 34.53.20 N 115.43.02 W; Kelso Dunes, San Bernardino, 2 ♂30/V/1999, Ballmer G.R. (UCR); Keen Camp, San Jacinto Mountains, 1 ♀ 31/V/1939, Smith Ray F.; Westwood Hills, Los Angeles, 1 ♂27/VII/1970, Linsley E.G. (USNM). **Utah:** Leeds Cyn. Washington Co., 1 ♂ 30/V/1973, Torchio P. & F. Parker (CNC).

### Crassomicrodus nigriceps

(Cresson, 1872)

http://species-id.net/wiki/Crassomicrodus_nigriceps

Fig. 10a–e

Crassomicrodus nigriceps (Cresson): Muesebeck 1927: 21–22 [Examined].
Microdus nigriceps Cresson 1872: 182.
Crassomicrodus fenestratus Viereck 1913: 558–559 [Examined]. Syn. n.

#### Holotype female.

 Collection Belfrage. Cat. No. 1637 (USNM)

#### Description female.

**Figure 10.**
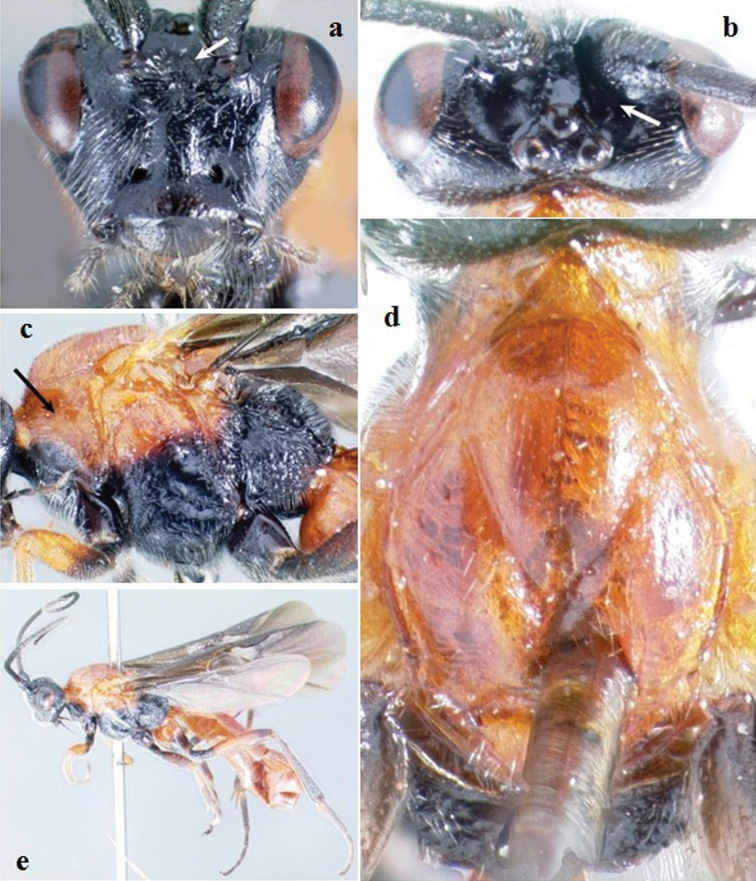
*Crassomicrodus nigriceps*. Female **a** anterior view of head, arrows indicate a median pyramidal-shaped elevation with two weakly defined tubercles **b** dorsal view of head, arrow indicates posterior surface of antennal sockets smooth **c** lateral view of mesosoma, arrow indicates pronotum smooth **d** dorsal view of mesosoma **e** female habitus.

 Body. Length. 6.05–9.50 mm. Color (Fig. 10e). Coloration of this species has a wide variation, there are specimens with the body totally dark to some areas yellowish-orange or yellow reddish; forewing infumate with a hyaline spot on the first submarginal cell, that occupies most of the space of the cell. Head (Fig. 10ab). Triangular in frontal view; face with weak longitudinal ridge dorsomedially; eye height/width = 1.48–1.50; eye height 0.67–0.68× inter-ocular distance; area between antennal sockets with a median pyramidal-shaped elevation and two weakly defined tubercles; frons excavated; posterior surface of antennal sockets smooth; groove between lateral ocelli smooth; median ocellus separated from lateral ocellus by smooth groove; gena not bulging, sometimes slightly bulging; malar space 0.51–0.64× as long as eye height; clypeus 1.88–2.21× wider than high; length of ventrolateral margin of clypeus similar to diameter of tentorial pit; antenna with 37–43 flagellomeres; setae at base of mandible distinctly longer than setae on rest of body surface. Mesosoma (Fig. 10cde). Pronotum smooth, sometimes strigose; lateral pronotal margins with weakly crenulate groove; notauli impressed; anterolateral edges of scutellum lacking small acute projection; scutellar disc convex with sparse setae from 0.11 to 0.12 mm in length; scutellar disc sloped posteriorly and rounded; lateral scutellar depression smooth, sometimes with punctures on the ventral margins; carinae of central metanotal area forming a triangular cell; propodeum reticulate rugose; subalar lobe separated from mesopleuron by narrow rugulose groove, width distinctly shorter than the subalar lobe; metapleuron reticulate rugulose. Legs. Inner spur of middle tibia 0.75–0.81× length of basitarsus; inner spur of hind tibia 0.59–0.62× length of basitarsus; metabasitarsus 1.21–1.29× length of tarsomeres III, IV, and V combined; hind tibia 2.04–2.22× longer than basitarsus; hind femur length 4.17–4.35× its maximum width. Wings. Forewing length/width = 2.70–2.76; stigma 3.57–3.85× longer than maximum width; forewing vein R1 0.62–0.69× as long as vein RS; vein RS straight; vein r arising before middle of stigma; second submarginal cell triangular, with petiole 0.07–0.22 mm long; vein M+CU distinctly pigmented throughout; hind wing length/width = 3.88–4.09; hind wing vein 1M 1.48–1.71× longer than 1r-m; hind wing with 6–8 hamuli. Metasoma. Apical width of petiole (tergum 1) 3.33–3.43× wider than basal width; minimum width of petiole 0.49–0.60× apical width; length of ovipositor sheath 0.17–0.26 mm.

#### Male.

Similar to female.

#### Host.

Unknown.

#### Distribution.

Colombia, Costa Rica, Cuba, El Salvador, Guatemala, Haiti, Honduras, Mexico, Puerto Rico, Dominican Republic, and the USA.

#### Diagnosis.

Distinguished from other *Crassomicrodus* species by the following combination of characters: area between antennal sockets with a median pyramidal-shaped elevation, head triangular in frontal view, face with a few sparce setae, setae at base of mandible distinctly longer than setae on rest of body surface, antenna with 37–43 flagellomeres, body usually with some areas yellowish-orange and wings infumate.

#### Remarks.

This species was described as *Microdus nigriceps* by Cresson (1872). Muesebeck (1927) considered it as valid species in the genus *Crassomicrodus*; however due to the wide range of color variation among specimens of this species, it is described to *Crassomicrodus fenestratus* as a different species, but the types of both species have affinity of characters. *Crassomicrodus nigriceps* is the most variable species, on coloration and measurements, that all species of *Crassomicrodus*. Our careful examination of each specimens allowed to group them in three sub-groups; the first sub-group formed by specimens from Texas (USA), of size 7.30 to 9.50 mm and the body color yellowish-orange or yellow reddish except head, propleuron, metapleuron, propodeum, ventral area of mesopleuron, and legs black; the coloration in mesosoma of this sub-group could vary to only propleuron black and some areas of legs yellowish-orange. The second sub-group is formed by specimens from the Yucatan peninsula (Mexico), of size 6.05 to 7.95 mm and the coloration blackish on all body, although the coloration could vary to only mesoscutum yellowish-orange or yellow reddish. The last sub-group is formed by specimens from Loggerhead Key island (Florida, USA), of size 6.80 to 7.30 mm and the coloration yellowish-orange or yellow reddish except head, propleuron, metapleuron and propodeum black, ventral area of mesopleuron and legs blackish, although the coloration on mesopleuron and legs could vary. The last sub-group is where the majority of the specimens that come from other places have more affinity.

#### Material examined.

Type ♀: *Microdus nigriceps*, collection Belfrage (USNM). Type ♀: *Crassomicrodus fenestratus* Porto Rico, C.W. Hooker (USNM). Homotypes ♀: **HONDURAS, [Siguatepeque]:** 30 km SE Siguatepeque, 11–12/VIII/1978, Chemsak J.A., E.G. Linsley & J.M. Linsley (CNC). ♂: **MEXICO, Oaxaca:** 23 miles S Matías Romero, 6/IV/1962, Parker F.D. (CNC). ♀, ♂: 27 miles SW Salina Cruz, 14/VII/1987, Wharton R. (TAMU). ♂: 63 miles E Tehuantepec, 16/IX/1967, 152 m., Painter E.M. & R.H. Painter (CNC). ♀: **NICARAGUA,** 1 miles SW Managua, 4/IX/1967, 2200 m., Painter R.H. & E.M. Painter (AEIC). *Other specimens examined*.- **COLOMBIA, Tolima:** Armero, 1 ♀ 30I–5II/1977, Peyton E.L. (USNM); 1 ♀ 26–30/1977, 1 ♂ 26–30/1977, Peyton & Suarez (HIC, USNM). **COSTA RICA, [Limón]:** Limon, 1 ♂ 15/II/1964, Evans H.E. (MCZ). **Guanacaste**: 10 km E Paseo Tempisque, 1 ♀ 30/VII/1990, Chamberlain W.F. (TAMU); Sector Santa Rosa; open field nr. road to Playa Naranjo, 1 ♀ 8–18/VI/1995, Dadelahi-Price & Zitani (ESUW). **CUBA,** Santiago de las Vegas 1 ♂ (USNM). **[Ciego de Avila]:** Baraguá, 1 ♀ 19/X/1927, Scaramuzza L.C. (USNM). **[Cienfuegos]:** Ciego Montero, Cruces, L.K., 1 ♂ XII/1917, Alayo P. (HIC); Baraguá, 1 ♀ 27/VIII/1927, Scaramuzza L.C. (MCZ). **Pinar del Rio:** San Vicente, 1 ♀ 26VII–5VIII/1939, Parsons C.T. (MCZ). **DOMINICAN REPUBLIC, [San Cristóbal]:** San Cristobal Prov., 1 ♂ 5/VIII/1967 (TAMU). **EL SALVADOR, [San Salvador]:** 6 miles W Quezaltepeque, 1 ♀ 2 ♂ 12/VIII/1963, 500 m., Cavagnaro D.Q. & M.E. Irwin (CAS); 6 miles W Quezaltepeque, 1 ♀ 15/VII/1963, 500 m., Cavagnaro D.Q. & M.E. Irwin (CAS); Sonzacate, 1 ♂ 25/VI/1958, Bottimer L.J. (HIC). **GUATEMALA. [Chiquimula]:** Mocá, Guatalón, 1 ♀ III–IV/1931, 1000 m., Bequaert J. (MCZ). **[Guatemala]:** Amatitlán, 1 ♀ 1 ♂ 6/VII/1947, 1219 m., Vaurie C. & P. Vaurie (AMNH); 6 miles N Amatitlan, 1 ♂ 29/XI/1972, Dasch B. & C. Dasch (AEIC). **Zacapa:** Rio Hondo, 3 ♀ VIII/1987, Sharkey M.J. (CNC, HIC). **HAITI,** Fond Parisien, 2 ♂ 11–18/II/1922, 18 m. (AMNH); Trou Caiman, 1 ♀ 1 ♂ 4/IX/1934, Bates M. (MCZ). **HONDURAS:** Nr. Corozal Brit., 1 ♀18/VII/1963, Porter C. (MCZ). **[Colón]:** Puerto Castilla, 1 ♂ 21/III/1924 (USNM). **El Paraíso:** Galeras, 1 ♂ 31/VII/1992, Stange L.; Yuscarán, 1 ♀ 4/VIII/1992, 840 m., Porter C. & L. Stange (FSCA). **La Paz:** La Paz, 1 ♂ 23/VI/1979, Chemsak J.A., A. Michelbacher, M. Michelbacher &W.W. Middlekauff (EMEC). **MEXICO, Baja California Sur:** 51 km W La Paz (km 51), 1 ♀ 26/VIII/1977, 275 m., Fisher E. & R. Westcott; 25 miles W La Paz, 1 ♂ 30/VIII/1959, Radford K.W. & Werner F.G. (CAS). **Campeche:** Champoton, 1 ♂ 8/VII/1964, Pallister J.C. & D. Pallister (AMNH). **Chiapas:** N. Chiapas, 3 km S Oaxaca Rte. 190, 1 ♂ 12/VIII/1962, 1524–1829 m., Milliron H.E. (CNC); 7 miles SE Soyalo, 1 ♂ 27/III/1953, Bechtel R.C. & E.I. Schlinger; Santo Domingo, 15 miles SE Simojovel, 1 ♀ 8–15/VII/1958, Smith R.F.; Suchiapa, 1 ♀ 18/VII/1957, Hurd P.D. (EMEC); Km 47 Tuxtla Gutierez-Venustiano Carranza, 1 ♂ 10/VII/1988, 480 m., Cadena A. & L. Cervantes (IBUNAM). **Coahuila:** 6 miles W Saltillo, 1 ♂ 14/VII/1972, 5200 ft., Dasch B. & C. Dasch (AEIC). **Colima:** Armeria, 1 ♂ 1/VIII/1954, Cazier M. y W. Gertsch Bradts (AMNH); 21 miles NW Manzanillo, 2 ♂ 30/VIII/1970, Wasbauer M.S. & J.S. Wasbauer, malaise trap; Playa de Oro Rd. NW Manzanillo, 1 ♀ 1 ♂ 31/VIII/1970, Wasbauer J.S. (EMEC). **Guerrero:** Xalitla, 1 ♂ 20/III/1959, 457 m., Evans H.E. & Anderson D.M. (CUIC). **Jalisco:** Chamela, 1 ♀ 26–30/IX/1985, Parker F.D. & T.L. Griswold; El Tuito, 1 ♂ 2/X/1985, Parker F.D. & T.L. Griswold; Guadalajara, 1 ♀ VIII/1962, Hull Frank M. (CNC); 22 km E El Grullo, 2 ♀ 17/VII/1989, Griswold T., 1200 m.; Chamela Res. Sta., 1 ♀ 24/VII/1986, Sanchez M- M.T. (HIC); Rio Santiago, 15 miles N Guadalajara, 1 ♂ 22/VII/1965, Evans H.E. (MCZ); 6 miles N Autlan, 1 ♂ 7/VII/1984, Shaffner, Woolley, Carroll & Friedlander (TAMU); Tequila, 1 ♀ 6/VII/1956, Dreisbach R. & K. Dreisbach (USNM). **Michoacán:** 11 miles E Apatzingan, 3 ♀ 20/VIII/1954, Linsley E.G., J.W. MacSwain & Smith R.F. (EMEC). **Morelos:** 3 miles N Alpuyeca, 1 ♀ 4/III/1959, 1036 m., Evans H.E. & Anderson D.M.; Las Estacas, 1 ♂ 6/IV/1959, 914 m., Evans H.E. (CUIC). **Nuevo León:** Apodaca, 1 ♀ 27/VII/1963, Howden H. & A. Howden (CNC); Carr. Miguel Aleman km 15, Apodaca, 1 ♂10/VIII/1984, Vera A. (CIBE-UANL). **Oaxaca:** Tehuantepec, 1 ♂ 23/XI/1972, Dasch B. & C. Dasch (AEIC); 23 miles S Matias Romero, 2 ♂ 14/VIII/1963, Parker F.D. & L.A. Stange (HIC, USNM); 23 miles S Matias Romero, 1 ♂ 22/IV/1962, Parker F.D.; El Camaron, 1 ♂ 24/IV/1962, Parker F.D. (USNM); **Puebla:** 12 miles N Chapulco, 1 ♀ 29/VII/1963, Caltagirone L.E. (EMEC). **Quintana Roo:** Felipe Carrillo Puerto, 4 ♀ 10–14/X/1986, 19.35 N 88.03 W, malaise trap; 1 ♂ 10–14/X/1986, Parker F.D., 19.35 N 88.03 W; 1 ♀ 2 ♂ 12–14/X/1986, Griswold T., 19.35 N 88.03 W (CNC). 25 km W Felipe Carrillo Puerto, 1 ♀ 15/X/1986, Parker F.D., 19.35 N 88.17 W; 3 km SW Puerto Morelos, 1 ♀ 1 ♂ 7/X/1986, Parker F.D.; Coba, 2 ♂ 5/X/1986, Griswold T., 20.36 N 87.35 W (CNC). Vallarta, 17 km W Puerto Morelos, 5 ♂ 6–8/X/1986, Griswold T., 2 ♂ 6–8/X/1986, Parker F.D.,20.50 N 87.00 W (CNC); 1 ♀ 6–8/X/1986, Griswold T. (HIC). **Sinaloa:** 34 miles N Los Mochis, 1 ♂ 27/VIII/1963, Parker F.D. & L.A. Stange (HIC); Mazatlán, 8 ♂ 15–20/VIII/1962, Evans H.E. (MCZ); Santa Ana, 1 ♀ 2/VIII/1985, Ekis G. (UCDC); Concordia, 1 ♂ 4/VII/1963, Parker F.D. & L.A. Stange (USNM). **Sonora:** Santa Ana, riv., 1 ♂ 4/VIII/1985, Ekis G. (UCDC); 19.4 miles S Estacion Llano, 2 ♀ 25/VIII/1964, Schlinger E.I., 700 m. ((USNM). **Veracruz:** Lake Catemaco, 1 ♂ 8–16/VIII/1960, Howden H.F. (CNC); Puente Nacional, 6 miles SE Rinconada, 1 ♂ 30/IX/1975, Chemsak J. (EMEC); Veracruz, 1 ♀ 1 ♂ 28VII–11VIII/1956, Dreisbach R. & K. Dreisbach (MSUC). **Yucatán:** Uxmal, 1 ♀ 16–18/VI/1959, Vaurie P. & C. Vaurie (AMNH); Mérida, 2 ♀ 22–25/VII/1962, Evans H.E. (ESUW); 8 miles E Merida, 1 ♂ 28/VI/1966 (HIC); Chichén Itzá, 1 ♂ 19/VII/1962, Evans H.E.; Mérida, 2 ♀ 1 ♂ 22–25/VII/1962, Evans H.E.; Uxmal, 1 ♀ 22/VII/1962, Evans H.E. (MCZ); 9 miles N Uxmal, 1 ♂ 1/VIII/1980, Schaffner, Weaver, Friedlander; Xmatkuil, Mérida, 1 ♀ 25–28/V/1996, Wharton & León, malaise trap (TAMU). 9 km N Teya Pueblo, 1 ♀ 1/IX/1999, 1 ♂ 12/X/1999, 2 ♂ 14/IX/1999, 1 ♀ 2/IX/1999, 1 ♀ 3 ♂ 3/VIII/1999, Suárez C. (UADY). **Tamaulipas:** Mesa de Llera, ca. Ciudad Victoria, 1 ♀ 1/VI/1977, Porter C. & A. Cerbone (FSCA). **PUERTO RICO,** Coamo Springs, 1 ♂ 1/VII/1915; Mayaguez, 1 ♀ 21–23/VI/1915 (AMNH); Lake Guanica, P.R., 5 ♂ 30/VI/1936, Dozier H.L.; Guanica, 2 ♀ 29/VI/1914, Smith; Lajas, 1 ♂ X–XI/1960, Cotte R. (USNM**)**. **USA, Arizona:** 1 ♂ 12/VIII/1974, Townes H. & M. Townes; Portal, 1 ♀ 17/VIII/1974, Townes H. & M. Townes (AEIC); Phoenix, 1 ♀ 2/VI/1942 (CAS); 5 miles E Nogales, Santa Cruz Co., 1 ♀ 1/IX/1970, Bohart G.E. & R.M. Bohart (CNC); 10 miles SW Patagonia, Santa Cruz Co., 1 ♀ 13/IX/1958, Cazier M.A. (EMEC). Madera Cyn., Santa Cruz Co, 1 ♀ 31/VII/1966, 1487 m., Kovacic C.R.; Box Cyn, Santa Cruz Co., 1 ♀26/VIII/1978, Meyer R.P. (UCDC). Wild Morning Glory, 2 ♀ 1957, Lochiel; W sl. Patagonia Mountains, 4 ♀ 9/VIII/1908, Butler G.D. & F.G. Werner (USNM). **Florida:** S. Miami 1 ♀ (MCZ). Isla Loggerhead Key, Dry Tortugas, 10 ♂ 1/IX/1961, 1 ♀ 4 ♂ 2/IX/1961, Weems H.V. Jr.; 2 miles NW Orange Spg., Putnam Co., 1 ♀ 13X–5XI/1975, Wiley J., malaise trap; Boca Chica Key, Monroe Co., 2 ♂11/VII/1971, Pierce W.H.; Clermont, Lake Co., 1 ♀ 29/VIII/1983, Nolfo V. (FSCA). Highlands Hamm. St. Pk. Fla., 1 ♂ 26/III/1963, Zeiger C.F.; Key Largo, 1 ♀ 2/V/1957, Weems H.V. (HIC). **Georgia:** St. Catherines Island, Liberty Co., 1 ♂ 22/V/1973, Rozen J.G. (AMNH). **Louisiana:** Gilliam, 61 ♂ /IX/1907, Bishopp F.C. (USNM). **North Carolina:** Jacksonville, Onslow Co., 1 ♀ 3/IX/1963, Bohart G.E. (CNC). **Texas:** Austin, 1 ♂ 26/VI/1922 (AMNH); 1 ♀ X/1899 (MCZ); 1 ♂ 26/VI/1922, Cazier M.A. (KSUC); 1 ♀ IX/1926, Bishopp F.C. (USNM); Bentsen Rio Grande State Park, Hidalgo Co., 1 ♀ 6–8/VI/1983, Pulawski W.J. (CAS). Brownsville, Cameron Co., 1 ♂11/IV/1976, Bruce Tilden; Sullivan City, Hidalgo Co., 1 ♂ 10/IV/1976 (CAS); Salado Creek, Bexar Co., 1 ♂ 13/III/1952, Wawbauer M. (EMEC). Fleming Key, Monroe Co., 1 ♀ 7/VIII/1979, 1 ♂ 8/VIII/1979, Acree John A. & H.V. Weems Jr., 1 ♀ 8–9/III/1980, Williams H.E. & H.V. Weems Jr. (FSCA). Valley Botanical Garden, McAllen, Hidalgo Co., 1 ♂ 12/VII/1980, 1 ♂ 23/VIII/1980, Porter C.C. (FSCA). Valley State Park, Bentsen Rio Grande, Hidalgo Co., 1 ♂ 4/VI/1982, 1 ♂ 1/VI/1979, 3 ♂ 11/VI/1982, 3 ♂14/VI/1982, 4 ♂ 16/VI/1983, 1 ♀ 2/VIII/1980, 1 ♀ 1 ♂ 30/V/1979, 2 ♀ 3 ♂ 31/V/1979, 1 ♂ 4/VI/1983, 1 ♀ 1 ♂ 6/VI/1982, Porter C. (FSCA). Valley State Park, near Mission, Bentsen Rio Grande, Hidalgo Co., 2 ♀ 6 ♂ 1–25/VIII/1980, 2 ♂ 10/VI/1981, 1 ♀ 1 ♂ 12/VI/1981, 1 ♂ 12/VIII/1983, 1 ♀ 2 ♂ 17/VI/1983, 1 ♂ 4/VI/1981, 2 ♂ 6/VI/1981, 1 ♂ 9/VI/1981, Porter C. (FSCA). McAllen Botanical Garden, Hidalgo Co., 1 ♀ 31/V/1982, 1 ♂ 31/VI/1982, Porter C. (FSCA). McAllen Botanical Garden, McAllen, Hidalgo Co., 1 ♀ 1/VII/1985, 1 ♀ 15/VI/1985, 1 ♀ 2/VII/1985, 1 ♀ 20/VI/1984, 1 ♀ 30/VI/1985, 1 ♀ 6/VII/1983, Porter C. (FSCA). San Antonio, 1 ♀ 11/IV/1942, Melander A.L.; Wharton, Wharton Co., 1 ♂ 24/VI/1917 (MCZ). Dallas, 1 ♀ 14/IX/1905, Jones C.R.; Galveston [Galveston Co.], 1 ♂ 29/VII/1924, Tretter; Garrett, 1 ♂ 21/VII/1908, Tucker E.S.; Plano [Collin Co.], 2 ♀ 1 ♂ X/1907, E.S. Tucker. Paris [Lamar Co.], 1 ♀ 27/VIII/1905, 1 ♂ 26/VIII/1905, F.C.Bishopp. Wolfe City, 1 ♀ 20/V/1907, 1 ♂ 4/VI/1909, F.C. Bishopp; Victoria, 1 ♂ 25/IX/1906, Crawford J.C.; Victoria, Victoria Co., 1 ♂ 25/VI/1917; Waco, 1 ♀ 17/II/1939, Jones C.R. (USNM).

### Crassomicrodus nigrithorax

Muesebeck, 1927

http://species-id.net/wiki/Crassomicrodus_nigrithorax

Fig. 11a–e

Crassomicrodus nigrithorax Muesebeck 1927: 17–18 [Examined].

#### Holotype female.

 Colorado [USA]. Cat. No. 28694 (USNM)

#### Description female.

**Figure 11.**
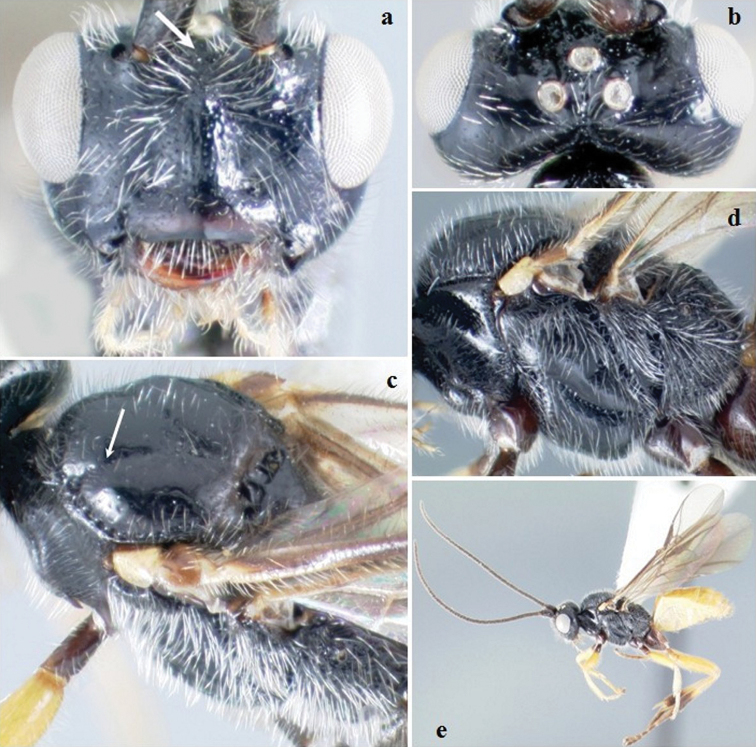
*Crassomicrodus nigrithorax*. Female **a** anterior view of head, arrows indicate a median transverse elevation with two weakly defined tubercles **b** dorsal view of head **c** dorsal view of mesosoma, arrow indicates notauli impressed **d** lateral view of mesosoma **e** female habitus.

Body. Length. 3.95–5.35 mm. Color (Fig. 11e). Integument black except yellowish as follows, three-quarter of the basal area of mandible, tegulae, femora, three-quarter of the basal area of hind tibia, fore and middle tibia, fore and middle tarsomeres, and metasoma; ocelli translucent yellow; eyes silver or blackish; wing veins clear brown; forewing slightly infumate with a hyaline spot on the first submarginal cell that is similar in size to the parastigma. Sometimes hind coxa and trochanters yellowish-orange and/or fore and middle femora, apical area of tibia, and fore and middle tarsomeres blackish, rarely tegula blackish. Head (Fig. 11ab). Transverse in frontal view; face with longitudinal ridge dorsomedially; eye height/width = 1.30–1.35; eye height 0.69–0.70× inter-ocular distance; area between antennal sockets with a median transverse elevation and two weakly defined lateral tubercles; frons excavated with a central groove almost foveolate, sometimes a pair of microfoveolate groove that diverge towards the ocellar area; posterior surface of antennal sockets smooth; groove between lateral ocelli microfoveolate; median ocellus separated from lateral ocellus by microfoveolate groove; gena bulging; malar space 0.38–0.47× as long as eye height; clypeus 2.67–2.91× wider than high; length of ventrolateral margin of clypeus similar to diameter of tentorial pit; antenna with 28–31 flagellomeres; setae at base of mandible slightly longer than setae on rest of body surface. Mesosoma (Fig. 11cde). Pronotum smooth except near of subpronope rugulose; lateral pronotal margins with weakly crenulate groove; notauli impressed; anterolateral edges of scutellum lacking small acute projection, sometimes slightly the projection; scutellar disc convex with sparse setae from 0.14 to 0.15 mm in length; scutellar disc sloped posteriorly and rounded; lateral scutellar depression rugose and foveolate on the margins and microfoveolate centrally; carinae of central metanotal area almost circular shaped; propodeum reticulate rugulose more pronounced on lateral margins, anterolateral areas with abundant setae; subalar lobe separated from mesopleuron by narrow rugulose groove, width distinctly shorter than the subalar lobe; metapleuron reticulate rugulose or punctulate, more pronounced on ventral half. Legs. Inner spur of middle tibia 0.89–0.95× length of basitarsus; inner spur of hind tibia 0.63–0.76× length of basitarsus; metabasitarsus 1.15–1.26× length of tarsomeres III, IV, and V combined; hind tibia 1.92–2.16× longer than basitarsus; hind femur length 3.23–3.38× its maximum width. Wings. Forewing length/width = 2.46–2.60; stigma 2.95–3.08× longer than maximum width; forewing vein R1 0.65–0.68× as long as vein RS; vein RS sinuate; vein r arising before middle of stigma; second submarginal cell triangular, with petiole 0.03–0.12 mm long; vein M+CU distinctly pigmented throughout; hind wing length/width = 3.45–3.58; hind wing vein 1M 1.83–1.97× longer than 1r-m; hind wing with 3–4 hamuli. Metasoma. Apical width of petiole (tergum 1) 2.80–2.82× wider than basal width; minimum width of petiole 0.64–0.70× apical width; length of ovipositor sheath 0.19–0.22 mm.

#### Male.

Similar to female.

#### Host.

Unknown.

#### Distribution.

Mexico and USA.

#### Diagnosis.

 Distinguished from other *Crassomicrodus* species by the following combination of characters: area between antennal sockets with a median transverse elevation, head transverse in frontal view, grooves between ocelli microfoveolate, gena bulging, antenna with 28–31 flagellomeres, anterolateral edges of scutellum usually lacking small acute projection, head and mesosoma black, and metasoma yellowish.

#### Remarks.

This species is near to *Crassomicrodus apicipennis*, but differs in that *Crassomicrodus apicipennis* has areas of mesosoma yellowish-orange; anterolateral edges of scutellum has small acute projection; eye height 0.65–0.68× inter-ocular distance; malar space 0.48–0.55× as long as eye height; inner spur of middle tibia 0.76–0.83× length of basitarsus; and scutellar disc convex with sparse setae from 0.16–0.17 mm in length.

#### Material examined.

 Holotype ♀: Colo (USNM). Allotype ♂: Colo (USNM). Homotype ♀: **MEXICO, Guerrero:** 4 miles S Taxco, 8/VIII/1954, 1463 m., Chillcott J.G. (CNC). *Other specimens examined*.- **MEXICO, Baja California Sur:** Las Animas, Sierra Laguna, 1 ♂ 1 ♀12/X/1941, Ross & Bohart (CAS). **Guerrero:** 17 miles NW San Marcos, 1 ♂13/VII/1966, Wagner P.M. & P.K. Wagner (TAMU). **Jalisco:** Chamela, PT, 1 ♂ 4–9/VII/1993, Sharkey M.J. (CNC). **Morelos:** Yautepec, 1 ♀ 23/VII/1963, Parker F.D. & L.A. Stange (HIC). **Nayarit:** 18 miles NW Ixtlán del Río, 1 ♂25/VII/1966, Wagner P.M. & P.K. Wagner (TAMU). **Nuevo Leon:** 5 miles S Monterrey, 1 ♂1/VII/1963, Howden H. & A. Howden (CNC). **Oaxaca:** 27 miles SW Salina Cruz, 3 ♀ 14/VII/1987, Wharton R. (TAMU); 3 miles W Oaxaca, 1 ♂ 21/XI/1972, Dasch B. & C. Dasch (AEIC). **Puebla:** 11 miles SE Acatlan, 1 ♂10/VII/1952, Gilbert E.E. & C.D. McNeil; 5 miles E Tepexco, 1 ♂24/VIII/1977, 1250 m., Schlinger E.I. (EMEC); 3 miles N Petalcingo, 1 ♀ 2 ♂3/VIII/1963, Parker F.D. & L.A. Stange; Petalcingo, 1 ♀ 3/VIII/1963, Parker F.D. & L.A. Stange (USNM). **San Luis Potosí:** 6 miles S Ciudad de Valles, 1 ♂21/VII/1954, 61 m., Chillcott J.G. (CNC); Valles, 1 ♂21/VII/1946, Pallister J. & D. Pallister (AMNH). **Sinaloa:** Cutiacan, 1 ♂11/IX/1970, Bohart G.E. & R.M. Bohart (HIC). **Tamaulipas:** Est. Carboneros Guemez, 1 ♂8/IX/1988, Loyola J.C. (CIBE-UANL); 44 miles W Tampico, 1 ♀ 22/VIII/1967, Hevel Gary F. (USNM). **Tlaxcala:** 21 miles W Apizaco, 1 ♀ 20/VIII/1958, Howden H.F. (CNC). **Veracruz:** 10 miles SW Perote, 1 ♂ 27/VII/1974, Clark-Murria-Ashe & Schaffner (TAMU). **USA, Arizona:** 13 miles SW Apache, Cochise Co., 1 ♂13/VIII/1970, Rozen J.G. (AMNH); Santa Cruz Sycamore Cyn. 9 miles W Peña Blanca Lk., 7 ♀ 12 ♂12/VIII/1983, 1250 m., Anderson R. (CNC); Tucson Mountains, 1 ♀ 1 ♂ 16/VIII/1955, Butler G.D. (USNM). **California:** Big Flat, Coffee Creek, Trinity Co. 1 ♀21/VI/1934, Van Dyke E.C. (CAS); 6 miles W Tragedy Spr., Amador Co., 1 ♀16/VII/1960, Rice R.E.; Donner Pass, 1 ♂ 1/VIII/1948, Townes H.M.G. & D. Townes (HIC); Westgard Pass, Inyo Co., 2 ♀ 16/V/1979, Bohart R.M.; 5 miles E Woodland, Yolo Co., 1 ♀ 10/IX/1970 (UCDC); Yuba, Sierra Co. 1 ♂ 6/VII/1962, Irwin M.E.; Quatal Canyon, NW corner Ventura Co., 1 ♀ 1 ♂ 9/V/1959, Powell J.; Davis, 1 ♂10/V/1960, Parker F.D. (USNM). **Colorado:** Colo 1 ♀ (HIC); **New Mexico:** Hatch, 1 ♀ 27/VIII/1974, Townes H. & M. Townes (CNC); Hatch, 1 ♀ 30/VIII/1974, Townes H. & M. Townes (AEIC). **Oregon:** Cave Jct., 1 ♂ 27/V/1978,Townes H. & M. Townes (AEIC). **Utah:** Strbry Daniel Pass, 1 ♀ 18/VI/1948, Townes H.M.G. & D. Townes (AEIC).

### Crassomicrodus oaxaquensis

Figueroa, Romero & Sharkey sp. n.

urn:lsid:zoobank.org:act:EF0FBCCA-7C2F-4AA6-8C0F-A4DAE53AEBF2

http://species-id.net/wiki/Crassomicrodus_oaxaquensis

Fig. 12a–e

#### Description female.

**Figure 12.**
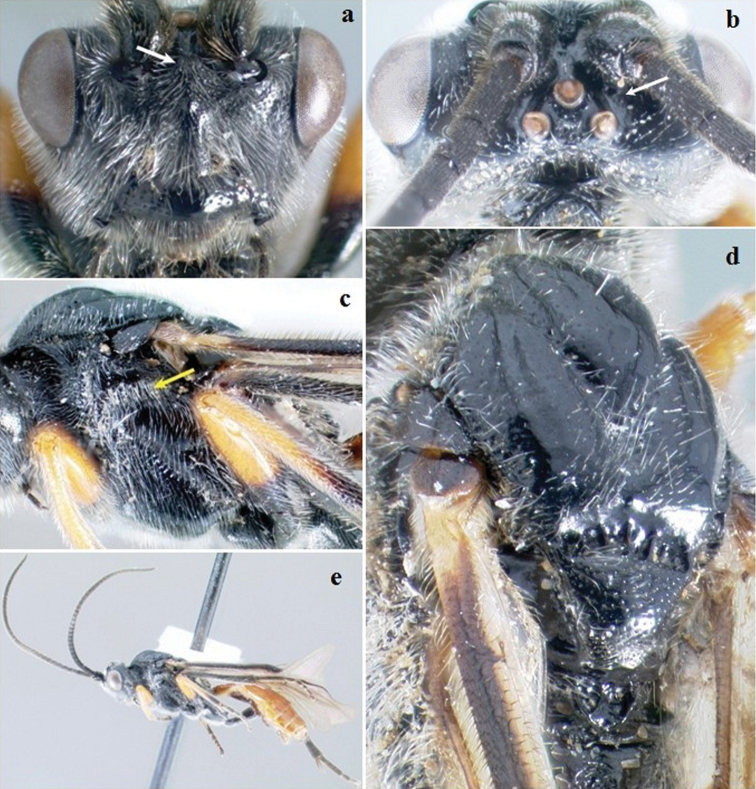
*Crassomicrodus oaxaquensis*sp. n. Female **a** anterior view of head, arrows indicate a median pyramidal-shaped elevation with two weakly defined tubercles **b** dorsal view of head, arrow indicates posterior surface of antennal sockets rugulose **c** lateral view of mesosoma, arrow indicates subalar lobe separated from mesopleuron by wide groove **d** dorsal view of mesosoma **e** female habitus.

Body. Length. 7.10–7.65 mm. Color (Fig. 12e). Integument black except yellowish-orange as follows, ocelli, fore tibia, two-thirds apical areas of fore and middle femur, two-thirds basal areas of middle tibia and metasoma; medial area of mandible yellow reddish; eyes silver; wing veins dark brown; forewing almost hyaline. Sometimes first metasomal tergite black, and the yellowish-orange of legs is reduced to only the apical area of fore and middle femora and apical area of fore tibia. Head (Fig. 12ab). Triangular in frontal view; face with weak longitudinal ridge dorsomedially; eye height/width = 1.34–1.45; eye height 0.60–0.62× inter-ocular distance; area between antennal sockets with a median pyramidal-shaped elevation and two weakly defined tubercles; frons excavated with a pair of microfoveolate grooves that diverge towards the ocellar area; posterior surface of antennal sockets rugulose; groove between lateral ocelli smooth; median ocellus separated from lateral ocellus by smooth groove; gena not bulging; malar space 0.55–0.58× as long as eye height; clypeus 2.25–2.44× wider than high; length of ventrolateral margin of clypeus almost similar to diameter of tentorial pit; antenna with 38–39 flagellomeres; setae at base of mandible slightly longer than setae on rest of body surface; face very setose. Mesosoma (Fig. 12cde). Pronotum punctate; lateral pronotal margins with weakly crenulate groove; notauli impressed; anterolateral edges of scutellum lacking small acute projection; scutellar disc slightly convex with sparse setae from 0.15 to 0.16 mm in length; scutellar disc sloped posteriorly and rounded; lateral scutellar depression rugulose and punctate; carinae of central metanotal area forming a triangular cell; propodeum reticulate rugulose with abundant sparse setae on lateral areas; subalar lobe separated from mesopleuron by wide rugose groove, width almost of similar size to subalar lobe; metapleuron reticulate rugulose in its ventral half and smooth or punctuate in its dorsal half. Legs. Inner spur of middle tibia 0.68–0.73× length of basitarsus; inner spur of hind tibia 0.58–0.66× length of basitarsus; metabasitarsus 1.25–1.32× length of tarsomeres III, IV, and V combined; hind tibia 2.33–2.38× longer than basitarsus; hind femur length 4.64–4.76× its maximum width. Wings. Forewing length/width = 2.57–2.76; stigma 2.91–3.33× longer than maximum width; forewing vein R1 0.65–0.70× as long as vein RS; vein RS sinuate; vein r arising before middle of stigma; second submarginal cell triangular, with petiole 0.08–0.09 mm long; vein M+CU distinctly pigmented throughout; hind wing length/width = 3.70–3.73; hind wing vein 1M 1.79–1.82× longer than 1r-m; hind wing with 5 hamuli. Metasoma. Apical width of petiole (tergum 1) 3.00–3.41× wider than basal width; minimum width of petiole 0.46–0.47× apical width; length of ovipositor sheath 0.22–0.26 mm.

#### Male.

Similar to female except metasomal tergite has the color dark brown, antenna with 39 or 40 flagellomeres, and hind wing with 4 or 5 hamuli.

#### Host.

Unknown.

#### Distribution.

Mexico.

#### Diagnosis.

Distinguished from other *Crassomicrodus* species by the following combination of characters: area between antennal sockets with a median pyramidal-shaped elevation, head triangular in frontal view, posterior surface of antennal sockets rugulose, face very setose, setae at base of mandible slightly longer than setae on rest of body surface, subalar lobe separated from mesopleuron by wide rugose groove, head and mesosoma black, and wings almost hyaline.

#### Remarks.

 This species is near to *Crassomicrodus jalisciensis*, but differs in that *Crassomicrodus jalisciensis* has areas of mesosoma yellowish orange, wings infumate, face without longitudinal ridge dorsomedially, and a median elevation between antennal sockets without defined lateral tubercles.

#### Etymology.

*Crassomicrodus oaxaquensis* is after Oaxaca, in reference to the known geographical distribution of the species.

#### Material examined.

 Holotype ♀: MEXICO, Oaxaca: Llano de las Flores, 15 miles NE Ixtlán de Juárez, 21/VII/1985, Woolley & Zolnerowich. Allotype ♂: same data as holotype. Paratypes 1 ♀, 2 ♂: same data as holotype. All types deposited in TAMU.

### Crassomicrodus olgae

Figueroa, Sharkey & Romero sp. n.

rn:lsid:zoobank.org:act:B4527E95-134E-4DD0-B8B3-00637C850D46

http://species-id.net/wiki/Crassomicrodus_olgae

Fig. 13a–e

#### Description female.

**Figure 13.**
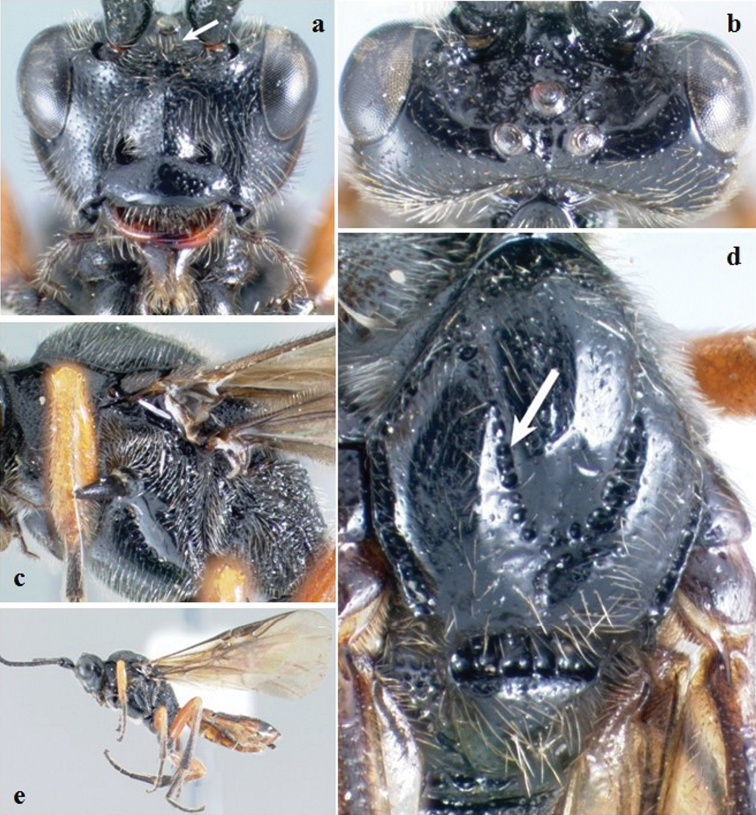
*Crassomicrodus olgae*sp. n. Female **a** anterior view of head, arrows indicate a median elevation in trapezoidal shape with two weakly defined tubercles **b** dorsal view of head **c** lateral view of mesosoma **d** dorsal view of mesosoma, arrow indicates notauli impressed **e** female habitus.

Body. Length. 6.70–7.08 mm. Color (Fig. 13e). Integument black except yellowish-orange as follows, medial area of mandible, femora, fore tibia, basal half of middle and hind tibia, and metasoma; ocelli translucent yellow; wing veins brown; forewing slightly infumate with a hyaline spot on the first submarginal cell that is similar in size to the parastigma. Head (Fig. 13ab). Transverse in frontal view; face with longitudinal ridge dorsomedially; eye height/width = 1.38–1.39; eye height 0.68–0.74× inter-ocular distance; area between antennal sockets with a median trapezoidal-shape elevation and two weakly defined tubercles; frons deeply excavated; posterior surface of antennal sockets smooth; groove between lateral ocelli slightly microfoveolate; median ocellus separated from lateral ocellus by smooth groove; gena distinctly bulging; malar space 0.54–0.59× as long as eye height; clypeus 2.08–2.19× wider than high; length of ventrolateral margin of clypeus similar to diameter of tentorial pit; antenna with 32 flagellomeres; setae at base of mandible distinctly longer than setae on rest of body surface. Mesosoma (Fig. 13cde). Pronotum with the pronotal groove reticulate rugulose and lateral areas smooth; lateral pronotal margins with weakly crenulate groove; notauli impressed; anterolateral edges of scutellum with slight acute projection, sometimes without projection; scutellar disc convex with sparse setae from 0.16 to 0.17 mm in length; scutellar disc sloped posteriorly and rounded; lateral scutellar depression with punctures centrally and foveolae in its margins; carinae of central metanotal area almost circular shaped; propodeum reticulate rugose, more pronounced on lateral margins; subalar lobe separated from mesopleuron by narrow rugulose groove, width distinctly shorter than the subalar lobe; metapleuron reticulate rugulose or foveolate in its ventral half and smooth in its dorsal half. Legs. Inner spur of middle tibia 0.85–0.92× length of basitarsus; inner spur of hind tibia 0.58–0.65× length of basitarsus; metabasitarsus 1.11–1.32× length of tarsomeres III, IV, and V combined; hind tibia 2.00–2.17× longer than basitarsus; hind femur length 3.50–3.85× its maximum width. Wings. Forewing length/width = 2.45–2.71; stigma 3.17–3.58× longer than maximum width; forewing vein R1 0.61–0.63× as long as vein RS; vein RS slightly sinuate; vein r arising slightly before middle of stigma; second submarginal cell triangular, with petiole 0.11–0.13 mm long; vein M+CU distinctly pigmented throughout; hind wing length/width = 3.36–3.76; hind wing vein 1M 1.83–1.85× longer than 1r-m; hind wing with 4 hamuli. Metasoma. Apical width of petiole (tergum 1) 2.88–3.18× wider than basal width; minimum width of petiole 0.48–0.55× apical width; length of ovipositor sheath 0.17–0.20 mm.

#### Male.

Similar to female except that male has 31–34 flagellomeres, fore and middle femora and tibia yellowish-orange, hamuli with 4 or 5 hooks; sometimes the petiole color blackish.

#### Host.

Unknown.

#### Distribution.

USA.

#### Diagnosis.

Distinguished from other *Crassomicrodus* species by the following combination of characters: area between antennal sockets with a median trapezoidal-shape elevation, head transverse in frontal view, gena distinctly bulging, groove between lateral ocelli slightly microfoveolate, anterolateral edges of scutellum usually with slightly acute projection, head and mesosoma black and wings slightly infumate.

#### Remarks.

 This species is near to *Crassomicrodus oaxaquensis*, but differs in that *Crassomicrodus oaxaquensis* has a triangular-shaped head, area between antennal sockets with a median pyramidal-shaped elevation, gena not bulging, and antenna with 38–39 flagellomeres.

#### Etymology.

 This species is named in honor of Olga Margot De la Rosa Reyes, mother of the first author.

#### Material examined.

 Holotype ♀: **USA, California:** Shingle, El Dorado Co., 22/V/1955, Burdick D.J. Allotype ♂: same data as holotype. Holotype and allotype deposited in EMEC. Paratypes: **California:** Tahoe National Forest, Pineland Drive 3.2 km S Tahoe City, Placer County, 1 ♂ 20/VII/1983, 1900 m., Davies Thomas W. (CAS); 4 miles S Railway Flat, Calaveras Co., 2 ♂ 19/V/1969, 853 m., Linsley E.G.; Shingle, El Dorado Co., 1 ♂ 22/V/1955, Burdick D.J. (EMEC); River Pines, Amador Co., 1 ♂ 26/IV/1975 (UCDC); Volcano, Amador Co., 1 ♂ 5/V/1957, Moore C.G. (USNM). **Utah:** 23 miles NE Logan, Cache Co., 1 ♀ 20/VI/1963, Toschi C.A. (EMEC).

### Crassomicrodus pallens

(Cresson, 1873)

http://species-id.net/wiki/Crassomicrodus_pallens

Fig. 14a–e

Crassomicrodus pallens (Cresson): Muesebeck 1927: 20.
Microdus pallens Cresson 1873: 53.

#### Holotype female.

 Illinois [USA]. Cat. No. 2746 (ANSP).

#### Description female.

Body. Length. 4.20–6.48 mm. Color (Fig. 14e). Integument yellowish-orange except ocelli translucent yellow; eyes black or silver; mandible apex and apical area of hind tibia and tarsomeres blackish. Sometimes propleuron black with metapleuron and propodeum blackish, rarely head and mesopleuron blackish. Wing veins dark brown; forewing infumate with a hyaline spot on the first submarginal cell that is similar in size to the parastigma. Head (Fig. 14ab). Triangular in frontal view; face without longitudinal ridge dorsomedially; eye height/width = 1.36; eye height 0.55–0.58× inter-ocular distance; area between antennal sockets with a median pyramidal-shaped elevation and two weakly defined tubercles; frons excavated with a pair of microfoveolate groove that diverge towards the ocellar area; posterior surface of antennal sockets smooth, rarely rugulose; groove between lateral ocelli smooth; median ocellus separated from lateral ocellus by smooth groove; gena not bulging; malar space 0.77–0.86× as long as eye height; clypeus 1.63–2.00× wider than high; length of ventrolateral margin of clypeus similar to diameter of tentorial pit; antenna with 29–34 flagellomeres; setae at base of mandible distinctly longer than setae on rest of body surface. Mesosoma (Fig. 14cde). Pronotum strigulose or reticulate rugulose; lateral pronotal margins with weakly crenulate groove; notauli impressed; anterolateral edges of scutellum lacking small acute projection; scutellar disc convex with sparse setae from 0.09 to 0.11 mm in length; scutellar disc sloped posteriorly and rounded; lateral scutellar depression smooth, rarely with punctures on the ventral margins; carinae of central metanotal area almost triangular shaped; propodeum reticulate rugulose; subalar lobe separated from mesopleuron by narrow rugulose groove, width distinctly shorter than the subalar lobe; metapleuron reticulate-rugulose. Legs. Inner spur of middle tibia 0.74–0.81× length of basitarsus; inner spur of hind tibia 0.61–0.72× length of basitarsus; metabasitarsus 1.02–1.12× length of tarsomeres III, IV, and V combined; hind tibia 2.22–2.39× longer than basitarsus; hind femur length 3.44–3.85× its maximum width. Wings. Forewing length/width = 2.46–2.55; stigma 3.00–3.44× longer than maximum width; forewing vein R1 0.47–0.57× as long as vein RS; vein RS not sinuate; vein r arising slightly before middle of stigma; second submarginal cell triangular, with petiole 0.05–0.09 mm long; vein M+CU distinctly pigmented throughout; hind wing length/width = 3.10–3.39; hind wing vein 1M 1.41–1.53× longer than 1r-m; hind wing with 4–5 hamuli. Metasoma. Apical width of petiole (tergum 1) 3.60–3.92× wider than basal width; minimum width of petiole 0.58–0.63× apical width; length of ovipositor sheath 0.20–0.33 mm.

#### Male.

Similar to female.

#### Host.

Unknown.

**Figure:**
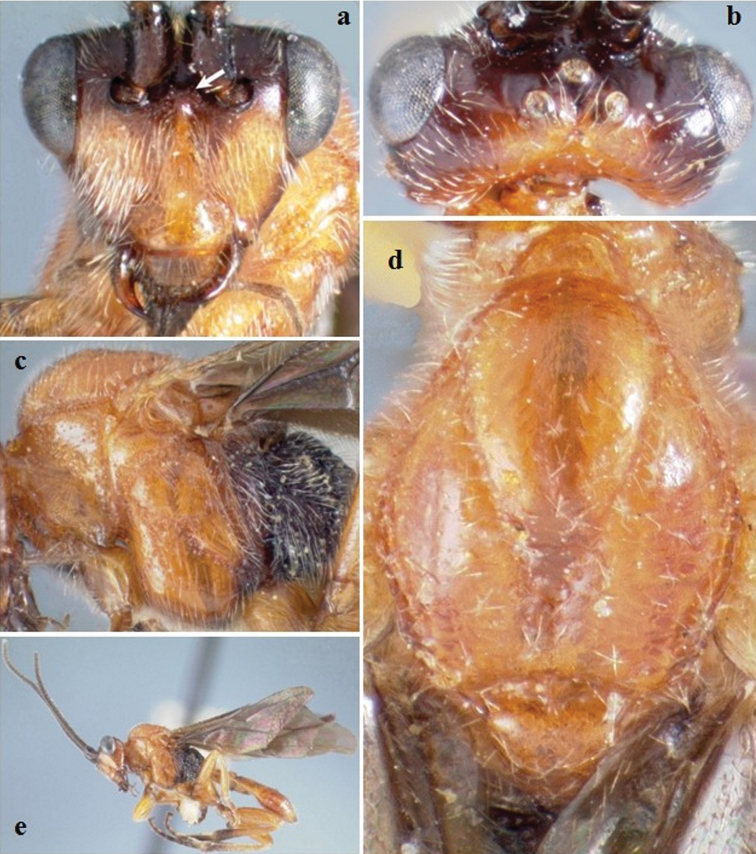
**Figure 14.** *Crassomicrodus pallens*. Female **a** anterior view of head, arrows indicate a median pyramidal-shaped elevation with two weakly defined tubercles **b** dorsal view of head **c** lateral view of mesosoma **d** dorsal view of mesosoma **e** female habitus.

#### Distribution.

Mexico and USA.

#### Diagnosis.

Distinguished from other *Crassomicrodus* species by the following combination of characters: area between antennal sockets with a median pyramidal-shaped elevation, head triangular in frontal view, malar space 0.77–0.86× as long as eye height, body length 4.20–6.48 mm, forewing vein R1 0.47–0.57× as long as vein RS, body usually yellowish-orange.

#### Remarks.

*Crassomicrodus pallens* resembles *Crassomicrodus divisus* in the shape of the head, but differs by the characters in the key. A few specimens of this species have the malar space shorter than eye height (0.77 times), specimens with this variation also have R1 less than 0.57 times as long as vein RS.

#### Material examined.

 Type: 1 ♀: Ill. (ANSP). Homotype: 1 ♀, **USA**, South Carolina: Hilton Head Island, 17/VII/1965, Howden H. & A. Howden (CNC).

*Other specimens examined*.- **MEXICO, Colima:** 21 miles N Manzanillo, 1 ♀ 25/VIII/1970, Wasbauer M.S. & J.S. Wasbauer (CNC). **Nayarit:** Arroyo Santiago, Nr. Jesus Maria, 2 ♀ 5/VII/1955 (CNC, EMEC). **Sinaloa:** 2.5 miles N Mazatlán, 1 ♀12/VIII/1970, Wasbauer M., malaise trap (CNC); 35 miles S Escuinapa, 1 ♂ 24/IV/1961, Howden & Martin (HIC). **Sonora:** Alamos, 2 ♀, 1 ♂ 5/IX/1970, Bohart R.M (UCDC); La aduana W Alamos, 1 ♀ 18/VIII/1964, Irwin M.E. (USNM); 5 miles E Navojoa, 1 ♀ 9/IX/1970, Bohart R.M. (UCDC). **Veracruz:** Minatitlán, 1 ♀ 1/II/1992, Osborn H. (USNM). **USA,** **Alabama:** Alabama, 2 ♀ 1980; Auburn, Lee Co., 1 ♀ 9/VI/1917; Tuskegee, 1 ♂ 22/VII/1930, Beamer R.H. (USNM). **Arkansas:** Washington Co. 1 ♀ 11/X/1955, Baker T.A. at light (USNM); Shoal Bay Rec. Area, 1 ♂20/V/1981, Dasch B. & C. Dasch (AEIC). **Connecticut:** East Hartford, 1 ♀ 13/VI/1947, Evans Howard E.; Riverbank, East Hartford, 1 ♀ 20/IX/1946, Evans Howard E. (CNC). **Florida:** 1 ♂(CNC). Tarpon Sprs., 1 ♂20/III/1950**,** Townes H.K.; Cedar Key, 1 ♂ 28/III/1985, Townes H. & M. Townes; Elfes, 1 ♀ 4/IV/1937, Franclemont J.G.; 5 miles NE Bronson, Levy Co., 1 ♂ 29/III/1986, LaSalle John (AEIC); De Funiak Springs, 1 ♂ 17–19/X/1914; Crestview, 1 ♂15–16/X/1914 (AMNH); Pine Hill Estates, Gainesville, 1 ♂4/X/1973, Weems H.V. Jr., malaise trap (CNC); Archbold Biol. Sta., Highlands Co., 1 ♀ 28VII10VIII/1987, Wahl D.B. (HIC); Fleming Key, Monroe Co., 2 ♀14/VIII/1979, Acree John A. & H.V. Weems Jr. insect flight trap; 1 miles W Interlachen, Putnam, 1 ♀, 5 ♂ 16/IV/1984, Stange L.A.; 2 miles N Holt, Okaloosa Co., 1 ♂28/X/1983, Stange L.A; Gainesville, Alachua Co., 1 ♂1/VIII/1977, 1 ♀ 31/VII/1977, Davis L.R. Jr.; Interlachen, Putnam Co., 2 ♂4/V/1986, Stange L.A.; Monteoca, Alachua Co., 1 ♀ 26/IX/1977, 1 ♀ 3/X/1977, Butler F. Jerry; Nokomis, Sarasota Co., 1 ♂9/I/1967; Palmdale, Glades County, 1 ♂25/VI/1972, Pierce W.H.; Pine Hill Estates, Gainesville, 1 ♀ 2/X/1973, 1 ♀ 20/IX/1973, 1 ♀ 29/IX/1973, 1 ♀ 30/IX/1973, Weems H.V. Jr., malaise trap; San Felasco Hammock, Alachua Co., 1 ♀ 17/III/1977, Fairchild G.B. & Weems H.V. Jr.; Suwanne Riv. St. Pk., Suwannee Co., 1 ♀ 13–25/IV/1977, Wiley J.R. (FSCA); San Felasco Hammock, Alachua Co., 1 ♀ 9–14/III/1977, Fairchild G.B. (UCDC); Arcadia, 1 ♂ 2/VII/1962, 1 ♂ 3/VII/1962, Krombein Karl V.; Alachua Co., 1 ♂4/VII/1955, Weems H.V.; Gainesville, 1 ♀ 28/VIII/1960, Stange L.A. (USNM). **Georgia:** 2 ♀ (USNM); Pine Mtn. Rabun Co., 1 ♂1/VIII/1957, 457 m., Chillcott J.G. (CNC); 10 miles N Waycross, Ware Co., 1 ♂ 27/VIII/1960, Marsh P.M.; Lakeland, 1 ♂ 18/IV/1940, Fattig P.W. (USNM). **Illinois:** 1 ♂ VIII (USNM); Havana, 1 ♂18/VIII/1912, Devil’s Hole; Meredosia, 1 ♂22/VIII/1917; St. Anne, 1 ♂22/VII/1935, Ross & DeLong (INHS); Carbondale, Jackson Co., 1 ♀ 28/IX/1956, Downey J.C. (USNM). **Indiana:** Forest Nursery, Jackson Co. 1 ♂ 23/IX/1938, Schnell R.L. (USNM). **Iowa:** Sioux City, 1 ♂ 15/VII/1927, Ainslie C.N. (USNM). **Kansas:** Lawrence, 1 ♀ 12/VI/1960, Menke A.S. (UCDC); 1 ♂ Baldwin, V, Bridwell J.C.; Lawrence vicinity, Douglas Co., 1 ♂ 29/VI/1962, Roberts R. (USNM). **Maryland:** Beltsville, 1 ♂ 23/V, Krombein K.V.; College Park, 1 ♂ 17/VIII/1914 (USNM); Crownsville, 1 ♀ 14/VII/1956, Krombein Karl V. (HIC). **Massachusetts** 2 ♂ (USNM); Truro 1 ♀, 1 ♂ 4/IX/1904, Morse A.P. (MCZ); Cabo Cod, 1 ♀ 6/IX/1939, Dreisbach R.R. (MSUC); Nantucket, 1 ♂ 7/IX/1909; Nantucket, 1 ♂ 8/IX/1926, Johnson C.W. (USNM). **Michigan:** Newago Co., 1 ♂ 13/VI/1940, Dreisbach R.R. (AEIC); Newago Co., 2 ♂ 30/VII/1944, Dreisbach R.R. (MSUC, USNM). **Minnesota:** Ft. Snelling, 1 ♀ 2/VIII/1923, Hertig A.T.; Ft. Snelling, 2 ♀ 28/VII/1922, Nichol A.A.; John Latsch St. Pk., S Minneiska, 1 ♀ 1/V/1951; Jordan, 1 ♀ 15/IX/1930, Talford H.S.; Jordan, sand area, 1 ♀ 13/VII/1923, Hertig A.T. (UMSP). **Missouri:** Columbia, 1 ♂ 29/IX/1964, Bayer L.G. (AEIC); Williamsville, 1 ♀ 1–16/VI/1969, Becker J.T., malaise trap (CNC); Columbia, 1 ♀ 20/VII/1967, Parker F.D. (UCDC); Taney Co., 1 ♀, 1 ♂13/IX/1944, Portman R.W. (UMRM); Columbia, 1 ♀ 22/VIII/1967, Parker F.D., malaise trap; Columbia, 1 ♀ 26/X/1931, Craig W.S. (USNM). **Nebraska:** Valentine Refuge, 1 ♀ 4/VI/1972, 1 ♂ 6/VI/1972, Townes H. & M. Townes (AEIC); Custer Co., 1 ♂ 21/VIII/1951, Dreisbach R.R. (MSUC). **New Jersey:** Palmyra, 1 ♀ 29/VIII/1933, Cazier M.A. (AMNH); Bergenfield, 1 ♂ VIII/1918?, Schott F.M.; Lucaston, 1 ♂ 12/IX/1902, Daecke E. (USNM). **New Mexico:** Hatch, 1 ♀ 29/VIII/1974, 1 ♀ 30/VIII/1974, Townes H. & M. Townes (AEIC). Riverton, 2 ♂ 5/IX/1948; Westville, 1 ♀ 12/IX/1897 (USNM). **New York:** Cold Springs Harb, 1 ♂25/VII, Melander A.L. (AEIC). **North Carolina:** Clinton, 1 ♂ 24/V/1951, Townes H. & M. Townes; Nags Head, 1 ♂, 1 ♀ 25/V/1948, Krombein K.V.; Smokemont, 1 ♂ 15/VIII/1947, Bullock & Dreisbach (AEIC); Fort Bragg, Cumberland Co., 1 ♀ 27IX/3X/1967, Birchim Jim D. (CAS); Cabo Hatteras mt. Buxton, 1 ♂14/VIII/1961, Howden H.; Highlands, 1 ♂4/VI/1957, 1158 m., Vockeroth J.R. (CNC). Jacksonville, Onslow Co., 1 ♂IX/1963; Kill Devil Hills, 2 ♂ 26/VI/1954, 1 ♀ 2/VII/1954, Krombein Karl V.; (HIC); Kill Devil Hills, 2 ♀, 6 ♂ 1/VII/1954, 1 ♀ 2/VII/1954, 1 ♀ 22/VI/1954, 2 ♂ 23/VI/1954, 1 ♀, 7 ♂ 26/VI/1954, 1 ♂ 28/VI/1954, 1 ♂ 3/VII/1954, 1 ♀, 8 ♂ 30/VI/1954, Krombein Karl V.; Raleigh, 1 ♂ 20/V/1937 (UMSP); Salvo, Dare Co., 1 ♂ 6/VIII/1958, Krombein (USNM); Southern Pines, 1 ♀ 3/V/1951, Howden H. & A. Howden; 1 ♀ 16/IX/1956, Krombein Karl V.; 1 ♂ 16/VIII (AEIC, USNM). **North Dakota:** 7 miles SE Sheldon, Ransom Co., 1 ♂5/VIII/1973, Powers J.R.; 11 miles W Walcott, Richland Co., 2 ♀ 23/V/1988, Powers J.R. (EMEC). **Oklahoma:** Lake Texoma 2 miles E Willis, 1 ♂ VI/1965, Bohart R.M. (UCDC); Cimarron River near Freedom, Woods Co. 1 ♀ 11/V/1984, Hevel G.F. & J.F. Hevel (USNM). **Pennsylvania:** Dauphin, 1 ♂ 6/VI/1909, Daecke E. (USNM); Philadelphia, 1 ♂, 1991?, Fox (USNM). **Rhode Island:** Providence 1 ♂ (USNM). **South Carolina:** Greenville, 1 ♂ 2/X/1941, 1 ♂ 8/X/1941, Townes H. & M. Townes (AEIC). **Tennessee:** Jefferson City, 2 ♀ 7/VII/1947, Valentine B.D. (CNC). **Texas:** Junction, Kimble Co., 1 ♀ 5/V/1986, Pulawski W.J. (CAS); Seagoville cerca Dallas, 2 ♀ XI/1944, Weyrauch (IMLA); Austin 1 ♂ (MCZ); Huntsville, Chartman 1 ♂; New Boston, 1 ♂ 17/X/1905, Bishopp F.C.; Pierce, 1 ♂ 22/IV/1907, alfalfa, Mitchell J.D. (USNM). **Virginia:** Ft. Humphreys, 1 ♂ 6/IX/1928, Mickel C.E. (UMSP); Barcroft, 1 ♂ 2/IX/1934, Bridwell J.C.; Falls Church, 1 ♀ 30/VIII/1923, Greene C.T.; Nelson Co., 1 ♀ 17/VIII/1927, Robinson W.; Rosslyn, 1 ♀ V/1929; Tazewell, 1 ♀ 9/VI/1915, Jackson L.O. (USNM). **Wisconsin:** Griffith St. Nursery, Wood Co., 1 ♂ 8/VI/1948, Shenefelt R.D. (AEIC).

## Supplementary Material

XML Treatment for Crassomicrodus

XML Treatment for Crassomicrodus apicipennis

XML Treatment for Crassomicrodus azteca

XML Treatment for Crassomicrodus clypealis

XML Treatment for Crassomicrodus costaricensis

XML Treatment for Crassomicrodus divisus

XML Treatment for Crassomicrodus fulvescens

XML Treatment for Crassomicrodus jalisciensis

XML Treatment for Crassomicrodus mariae

XML Treatment for Crassomicrodus melanopleurus

XML Treatment for Crassomicrodus muesebecki

XML Treatment for Crassomicrodus nigriceps

XML Treatment for Crassomicrodus nigrithorax

XML Treatment for Crassomicrodus oaxaquensis

XML Treatment for Crassomicrodus olgae

XML Treatment for Crassomicrodus pallens
